# Revealing the impact of lifestyle stressors on the risk of adverse pregnancy outcomes with multitask machine learning

**DOI:** 10.3389/fped.2022.933266

**Published:** 2022-12-13

**Authors:** Martin Becker, Jennifer Dai, Alan L. Chang, Dorien Feyaerts, Ina A. Stelzer, Miao Zhang, Eloise Berson, Geetha Saarunya, Davide De Francesco, Camilo Espinosa, Yeasul Kim, Ivana Marić, Samson Mataraso, Seyedeh Neelufar Payrovnaziri, Thanaphong Phongpreecha, Neal G. Ravindra, Sayane Shome, Yuqi Tan, Melan Thuraiappah, Lei Xue, Jonathan A. Mayo, Cecele C. Quaintance, Ana Laborde, Lucy S. King, Firdaus S. Dhabhar, Ian H. Gotlib, Ronald J. Wong, Martin S. Angst, Gary M. Shaw, David K. Stevenson, Brice Gaudilliere, Nima Aghaeepour

**Affiliations:** ^1^ Department of Anesthesiology, Perioperative and Pain Medicine, Stanford University, Palo Alto, CA, United States; ^2^ Department of Pediatrics, Stanford University, Palo Alto, CA, United States; ^3^ Department of Biomedical Data Science, Stanford University, Palo Alto, CA, United States; ^4^ Chair for Intelligent Data Analytics, Institute for Visual and Analytic Computing, Department of Computer Science and Electrical Engineering, University of Rostock, Rostock, Germany; ^5^ Department of Pathology, Stanford University, Palo Alto, CA, United States; ^6^ Department of Microbiology & Immunology, Stanford University, Palo Alto, CA, United States; ^7^ Baxter Laboratory for Stem Cell Biology, Stanford University, Palo Alto, CA, United States; ^8^ Department of Psychology, Stanford University, Palo Alto, CA, United States; ^9^ Department of Psychiatry & Behavioral Science, University of Miami, Miami, FL, United States; ^10^ Department of Microbiology & Immunology, University of Miami, Miami, FL, United States; ^11^ Sylvester Comprehensive Cancer Center, University of Miami, Miami, FL, United States; ^12^ Miller School of Medicine, University of Miami, Miami, FL, United States

**Keywords:** adverse pregnancy outcomes (APO), psychosocial and stress-related factors, prediction, multitask machine learning, immune states, single-cell

## Abstract

Psychosocial and stress-related factors (PSFs), defined as internal or external stimuli that induce biological changes, are potentially modifiable factors and accessible targets for interventions that are associated with adverse pregnancy outcomes (APOs). Although individual APOs have been shown to be connected to PSFs, they are biologically interconnected, relatively infrequent, and therefore challenging to model. In this context, multi-task machine learning (MML) is an ideal tool for exploring the interconnectedness of APOs on the one hand and building on joint combinatorial outcomes to increase predictive power on the other hand. Additionally, by integrating single cell immunological profiling of underlying biological processes, the effects of stress-based therapeutics may be measurable, facilitating the development of precision medicine approaches.

**Objectives:**

The primary objectives were to jointly model multiple APOs and their connection to stress early in pregnancy, and to explore the underlying biology to guide development of accessible and measurable interventions.

**Materials and Methods:**

In a prospective cohort study, PSFs were assessed during the first trimester with an extensive self-filled questionnaire for 200 women. We used MML to simultaneously model, and predict APOs (severe preeclampsia, superimposed preeclampsia, gestational diabetes and early gestational age) as well as several risk factors (BMI, diabetes, hypertension) for these patients based on PSFs. Strongly interrelated stressors were categorized to identify potential therapeutic targets. Furthermore, for a subset of 14 women, we modeled the connection of PSFs to the maternal immune system to APOs by building corresponding ML models based on an extensive single cell immune dataset generated by mass cytometry time of flight (CyTOF).

**Results:**

Jointly modeling APOs in a MML setting significantly increased modeling capabilities and yielded a highly predictive integrated model of APOs underscoring their interconnectedness. Most APOs were associated with mental health, life stress, and perceived health risks. Biologically, stressors were associated with specific immune characteristics revolving around CD4/CD8 T cells. Immune characteristics predicted based on stress were in turn found to be associated with APOs.

**Conclusions:**

Elucidating connections among stress, multiple APOs simultaneously, and immune characteristics has the potential to facilitate the implementation of ML-based, individualized, integrative models of pregnancy in clinical decision making. The modifiable nature of stressors may enable the development of accessible interventions, with success tracked through immune characteristics.

## Introduction

The adverse pregnancy outcomes (APOs) that we will focus on in this work - including early gestational age (GA) at delivery (a continuous surrogate for preterm birth), gestational diabetes, severe preeclampsia (severe pree), or preeclampsia superimposed on hypertension (superimposed pree) - are associated with severe short- and long-term consequences for both the mother and infant (1–4), underscoring the importance of management and prevention (5–9). Currently, however, highly efficacious therapeutic options to prevent APOs are lacking, highlighting the critical clinical need to identify actionable risk targets and prevent their development (10). In this context, epidemiological evidence has linked psychosocial and stress-related factors (PSFs) to APOs (11–16). Such PSFs are defined as internal or external stimuli that can induce biological changes (17) and are threats perceived by an individual that can result in psychopathological symptoms (18, 19). Importantly, the complexity of PSFs can be conveniently captured through questionnaires (11). In this work, we use the previously studied self-filled Dhabhar Quick-Assessment Questionnaire for Stress and Psychosocial Factors (DQAQ-SPF), that is designed to quantify commonly studied factors (perceived pregnancy risks, life stress, support) and factors not traditionally studied in the context of APOs (concerns about health, pain, and sleep) efficiently (12).

The connection between APOs and PSFs has been observed in multiple studies. For example, PSFs have been associated with spontaneous preterm birth (PTB), while protective psychological factors such as a robust support network decrease PTB risk (12, 20, 21). Similarly, hypertensive disorders of pregnancy are more likely to occur when women experience high levels of psychosocial stress, depression, and/or anxiety (11, 16, 22, 23). Moreover, recent studies have shown that chronic and financial stressors are strongly associated with gestational diabetes (24). In addition, maternal exposure to bereavement-related stress increases the risk of diabetes in a fetus and child later in life, thereby extending the adverse effects of PSFs to a child's well-being (13, 23). Given this strong connection between PSFs and APOs, PSFs are significant and accessible targets to determine risk for, clinically manage, and potentially prevent APOs.

Even though there is a strong connection between PSFs and APOs, PSFs are multifaceted, and their relations to individual APOs can be complex. This is further complicated by the difficulties of studying APOs in isolation, as they are tightly interrelated and integrated into an intricate biological environment. For instance, a common reason for preterm delivery is the development of preeclampsia early during pregnancy (21). In addition, gestational diabetes has been linked to increased risks of PTB and preeclampsia (25, 26). Furthermore, pre-pregnancy comorbidities and risk factors (RFs) create an unfavorable pathophysiological state during pregnancy that is associated with the development of APOs. For example, hypertensive disorders of pregnancy are correlated with a higher body mass index (BMI) and a history of diabetes (11, 27); similarly, gestational weight gain in women with gestational diabetes is associated with hypertensive disorders, preeclampsia, and PTB (26). The risk for PTB also increases in pregnancies with pre-existing systemic comorbidities such as diabetes or hypertension (28). This interconnectedness of APOs, as well as their relations with various risk factors, warrants the investigation of multiple APOs simultaneously on an integrated level, moving towards a systems approach for understanding the relations among pathophysiological risk factors (RFs; including diabetes, hypertension, and pre-pregnancy BMI), APOs, and PSFs.

**Figure 1:**
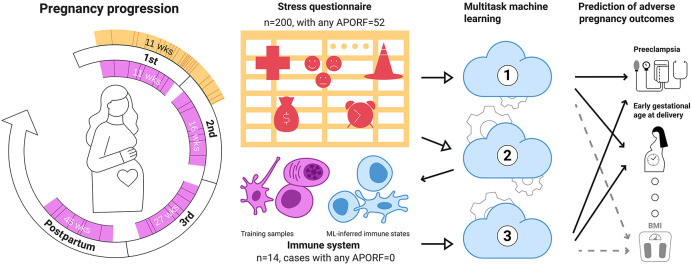
Multitask machine learning for the joint modeling of adverse pregnancy outcomes, stress, and biology. A stress questionnaire assessing psychosocial and stress-related factors (PSFs) related to perceived pregnancy risks, emotions and feelings, and life stress was administered during the first trimester (orange) to 200 women. 52 of these women experienced at least one adverse pregnancy outcome (solid lines) or pathophysiological risk factor (dashed lines). Distribution of gestational age at questionnaire completion. In most instances (>95%), the questionnaires were completed before 21 weeks of gestation. The median gestational age when completing the questionnaire was 11 weeks. In addition, patients were followed throughout pregnancy, and immunological data (designated in pink) were obtained during the first (median = 11 weeks), second (median = 16 weeks), and third trimester (median = 27 weeks), as well as at 6 weeks postpartum (median = 45 weeks). Model 1: Based on these data, a multitask approach was used to simultaneously model multiple adverse pregnancy outcomes (in bold) as well as risk factors (RFs; Normal font) based on PSFs, building upon their interrelatedness to improve predictions. Model 2: PSFs were used to predict immune system states (pink cells) illustrating a link between stress factors and biological functions. Finally (Model 3), connections among APOs, PSFs and the immune system were investigated by deriving immune states for our wider dataset from PSFs (blue cells) for predicting APOs. Overall, our results point towards a tight relationship between adverse pregnancy outcomes, stress and immune biology. Importantly, they suggest that several PSF categories can be jointly informative in modeling APOs pointing towards potential easily accessible stress-based interventions for APOs.

In this work, we modeled the connection of PSFs to APOs and pathophysiological RFs (APORFs) jointly to identify key associations underlying the holistic nature of their interrelatedness. To this end, we used multitask machine learning (MML), a powerful method to model interrelated and comparably infrequent outcomes simultaneously (29–31). For this, we leveraged a dataset of 200 pregnant women with various APORFs, for which PSFs were assessed during the first trimester in an extensive questionnaire. We modeled and predicted APORFs for these patients based on PSFs using MML. For an overview of this experiment, see Figure 1, **Model 1**.

Furthermore, based on a smaller dataset, we analyzed the maternal circulating immune system (in peripheral blood) to explore the biological underpinning of identified associations with the aim of revealing potential targets for measurable and accessible interventions. In particular, for a subset of 14 women, we modeled the connection of PSFs to the maternal immune system by building corresponding machine learning (ML) models from an immune data set measured using mass cytometry time of flight (CyTOF). We used these models predicting immune characteristics from PSFs to clarify potential joint pathways underlying PSFs and immunobiology. This model was then used to generate missing immune system data for patients diagnosed with APOs and successfully predicted APORFs from these in-silico engineered immune system characteristics using MML, highlighting the links among stress, the immune system, and pregnancy. An overview of the corresponding experiments is illustrated in Figure 1 **(Model 2 and 3)**.

## Materials and methods

### Data

#### Study cohort and stress questionnaire

Data was accumulated from the Prematurity Research Center Cohort (PRCC), established in 2011 at Stanford University Hospitals and Clinics. Women were enrolled prior to 12 weeks gestation. An extensive self-filled stress questionnaire, the Dhabhar Quick-Assessment Questionnaire for Stress and Psychosocial Factors (DQAQ-SPF) previously employed in the context of preterm birth (12), was provided during the first trimester of pregnancy to assess chronic stress, life events, emotions, personality, and sleep (Supplementary Tables S3, S4). In addition, perceived pregnancy risk was assessed with questions regarding concern for birth complications (“Some women may say that their risk for having birth complications like an early delivery, low birth weight or pregnancy complications (not birth defects) is low, average, or high. Think about yourself compared to most other pregnant women your age.”) and birth defects (similar to previous question). Health-related questions were based on the Medical Outcomes Study 36-Item Short-Form Health Survey, administered during the enrollment visit, at a median of 11 weeks gestation. Participants rated items on an eight-point scale or provided “yes/no” answers regarding specific life experiences; they were allowed to answer “not applicable” or skip questions. Demographic data collected included race/ethnicity, education, and age (also see Supplementary Table S1).

79 stressors, including binary, categorical, ordinal, and numeric variables, were selected for prediction. First, we removed all non-numeric variables. Then, we added certain text-based variables by parsing and converting them to numbers, including, for example, “preferred bedtime.” We then removed features with only one unique and/or missing values. Life stress from 41 to 50 (“How stressful was your life between the ages of 41–50?”) was dropped from our analysis, as our entire patient population was younger than 41. The initial cohort had 283 patients; however, 83 patients without preeclampsia information were removed from our dataset. Analysis was based on the remaining 200 pregnant women; 18 with hypertension, 11 with diabetes, 29 with gestational diabetes, 19 with preeclampsia (6 superimposed, 13 severe), 17 preterm births; 52 women had one or more of these APOs (Supplementary Table S1, Supplementary Figure S3). All maternal adverse pregnancy outcomes were obtained from electronic medical records; coordinators reviewed medical charts of all enrollees for the outcomes of interest.

#### Single-cell immune measurements

The analysis leveraged existing immunological data collected in a prospective study, immune data was collected from pregnant women seen at the obstetrics clinics of the Lucile Packard Children's Hospital at Stanford University for prenatal care (32). Women were included if they were at least 18 years of age and in their first trimester of a singleton pregnancy. Four peripheral blood samples were collected - during the first trimester (7 to 14 weeks), second trimester (15 to 20 weeks), third trimester (24 to 32 weeks), and 6-week postpartum. Samples were analyzed using mass cytometry time of flight (CyTOF) for single-cell characterization of the immune system (32). A total of 984 immune features were extracted from each whole blood sample using a 41-parameter assay (antibody panel), including the frequencies of 24 major innate and adaptive immune cell subsets, their endogenous intracellular signaling activities (phosphorylated = activated signaling proteins of the MyD88, Jak/STAT, mTOR, and NFkB signaling pathways), and the signaling capacities of each cell subset to respond to a series of receptor-specific immune challenges [15-min stimulation with lipopolysaccharide (LPS), interferon-a (IFN-α), and a cocktail of interleukins (IL-2, IL-6)]. All timepoints of a patient were analyzed simultaneously to minimize batch effects.

### Statistical analyses

#### Multitask prediction of adverse pregnancy outcomes and risk factors

**Figure 2:**
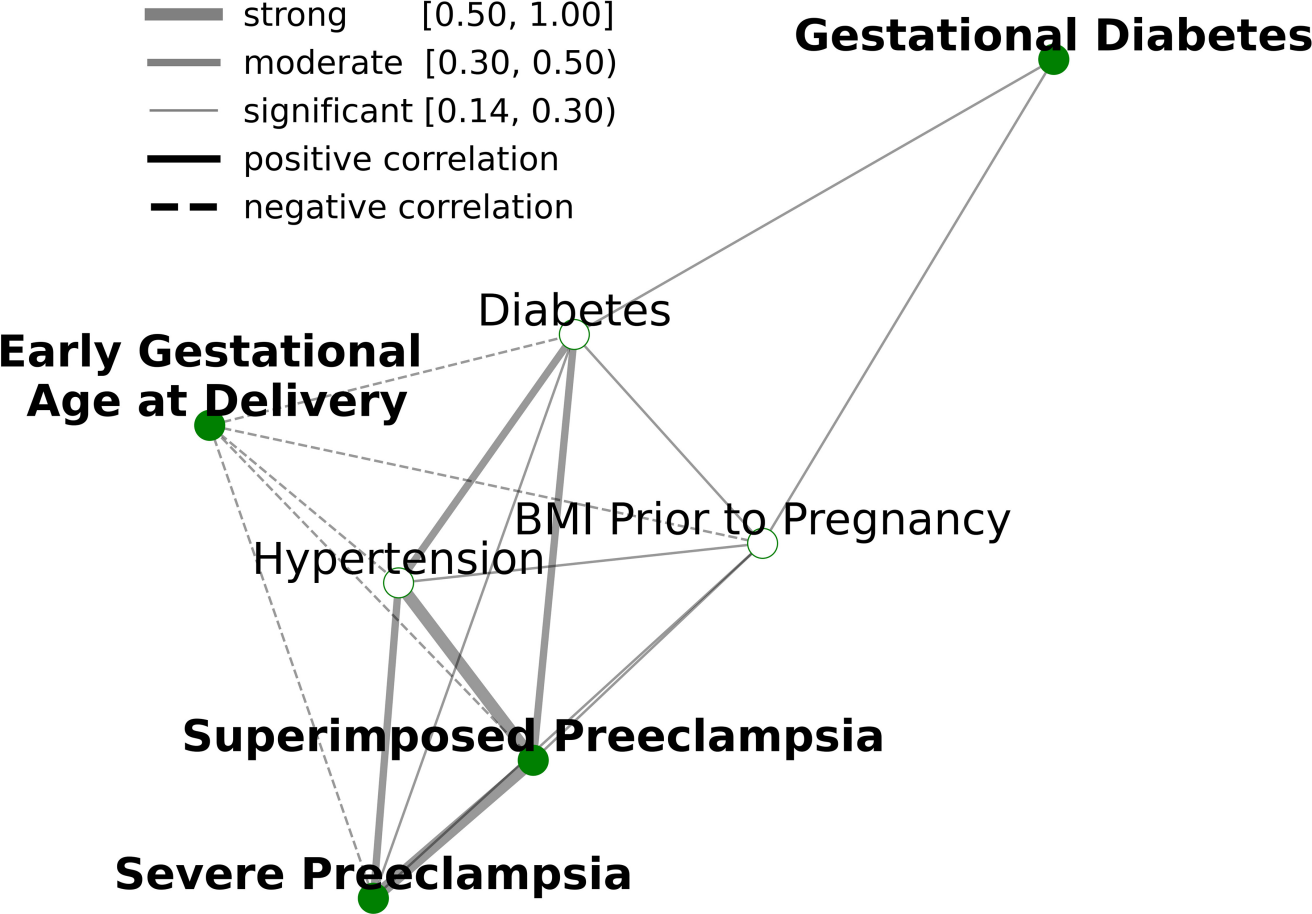
Relationships between APORFs during the first trimester. Green nodes represent APOs while white nodes represent RFs present before conception. Edges represent the strength of the Spearman correlation between two outcomes, with green representing strong, orange representing moderate, and grey representing weak correlations, as defined in Akoglu 2018. “Pree” is short for preeclampsia. APORFs are strongly correlated with one another, laying the foundation to jointly model these outcomes in a MML setting for improved predictive performance.

**Figure 3:**
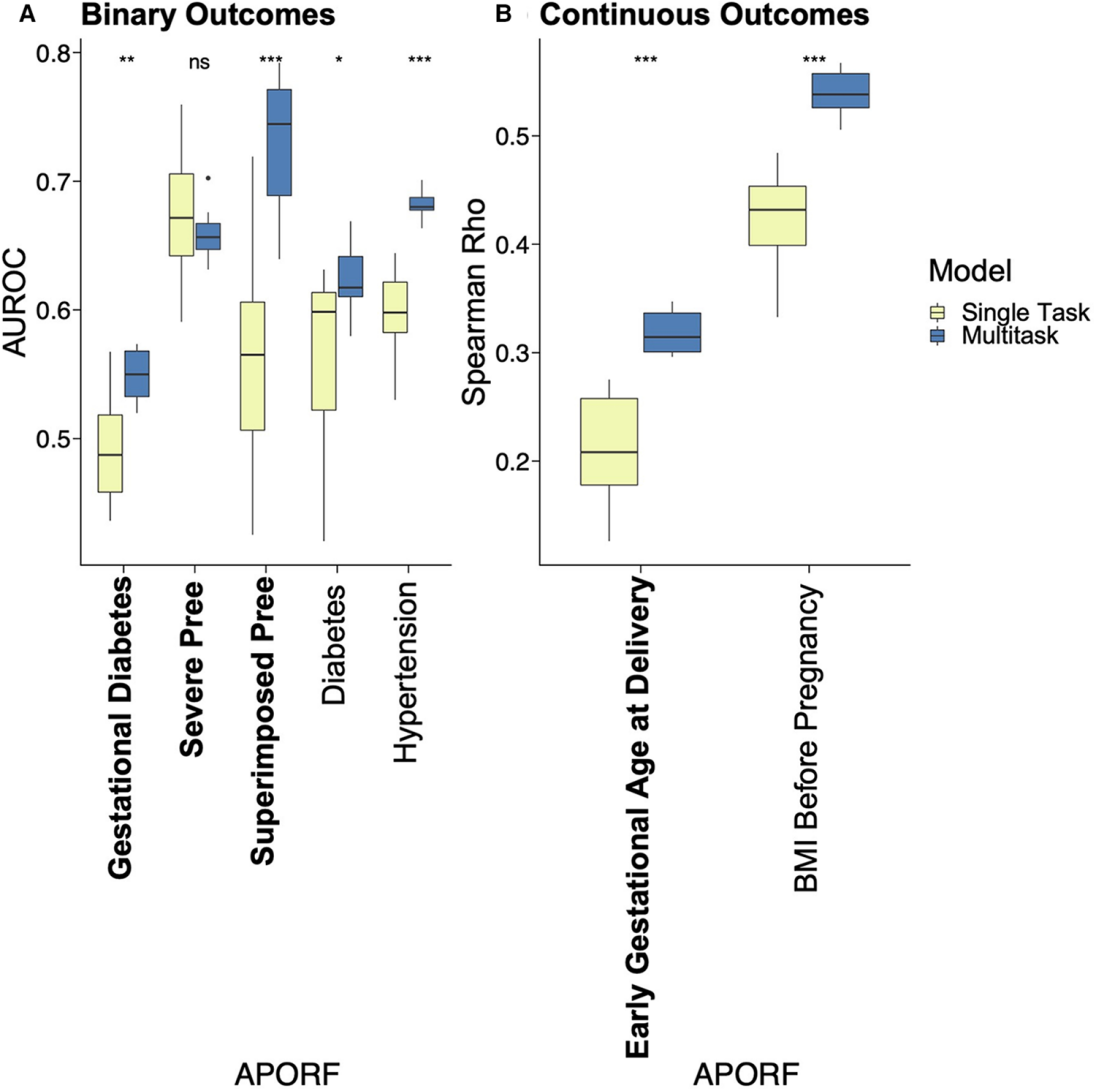
Performance comparison of single task vs. MML for predictive models of APOs. APOs are bolded, and pathophysiological RFs are unbolded. Results of multitask models are blue, and single task models are yellow. Asterisks represent significant differences by the Wilcoxon test between single task and multitask approaches (ns: *p* > 0.05, *p <= 0.05, **p <= 0.01, ***p <= 0.001, and ****p <= 0.0001). “Pree” is an abbreviation for preeclampsia. With one exception where no significant prediction differences are recorded, multitask models significantly outperformed single task models in all cases, emphasizing the interrelatedness between APORFs and successful joint modeling.

The employed multitask approach builds upon the interconnectedness of APOs (Figure 2) and simultaneously models highly related risk factors (RFs) to further exploit joint combinatorial outcomes for increased power. Multitask neural networks and single task approaches were compared across the prediction of gestational diabetes, severe preeclampsia, superimposed preeclampsia, and GA at delivery, as well as diabetes, hypertension, and BMI prior to pregnancy. GA at delivery and BMI prior to pregnancy were numeric variables; the rest were binary. The only mild case of superimposed preeclampsia was removed because mild and severe preeclampsia differ in risk and management. Preeclampsia with and without underlying hypertension were treated as two binary variables due to different underlying pathology. The multitask neural network architecture consisted of a feature embedding layer followed by two fully connected hidden layers of 150 hidden units each that then fed into outcome-specific modules of 150 hidden units each that terminated in separate regression or classification heads. Batch normalization and Rectified Linear Unit (ReLU) activation were employed at each hidden layer. Deep learning models were trained for a maximum of 35 epochs with early stopping with a patience configuration of 1 epoch. The classification criterion used for loss was binary cross-entropy loss, and mean squared error was used for regression outputs (scaled such that numerical values did not overpower the binary cross-entropy loss). Deep learning models were trained using the Adam optimizer with a batch size equivalent to the length of the training set. The single task neural network had the same architecture as the multitask network except for a single regression or classification output. Both single task and multitask deep learning models were trained using repeated stratified K-Fold splitting with 5 folds to evaluate model variance. For Figure 3, the area under the receiver operator characteristic (AUROC) was used to measure model performance of binary variables, and Spearman's rank correlation coefficient (rho) was used to measure model performance of numeric variables. Model performance was evaluated on the test set predictions collected over the repeated K fold procedure. The Wilcoxon test was used to classify the level of significance of single task vs. multitask models.

#### Cluster analysis of psychosocial and stress-related factors

An interdependency network of PSFs was created to understand their interrelatedness (Supplementary Figure S5). For this, a correlation matrix between all these factors was calculated using Spearman correlation (omitting undefined values). Analogously to previous work (12), factors were then grouped into 15 clusters using the K-Means algorithm on the corresponding correlation vectors. A two-dimensional embedding for placing each factor was calculated using the t-distributed stochastic neighbor embedding (33) on the absolute values of the correlation vectors associated with each factor. These clusters of psychosocial and stress factors were then analyzed in the context of their impact on APOs.

**Figure 4:**
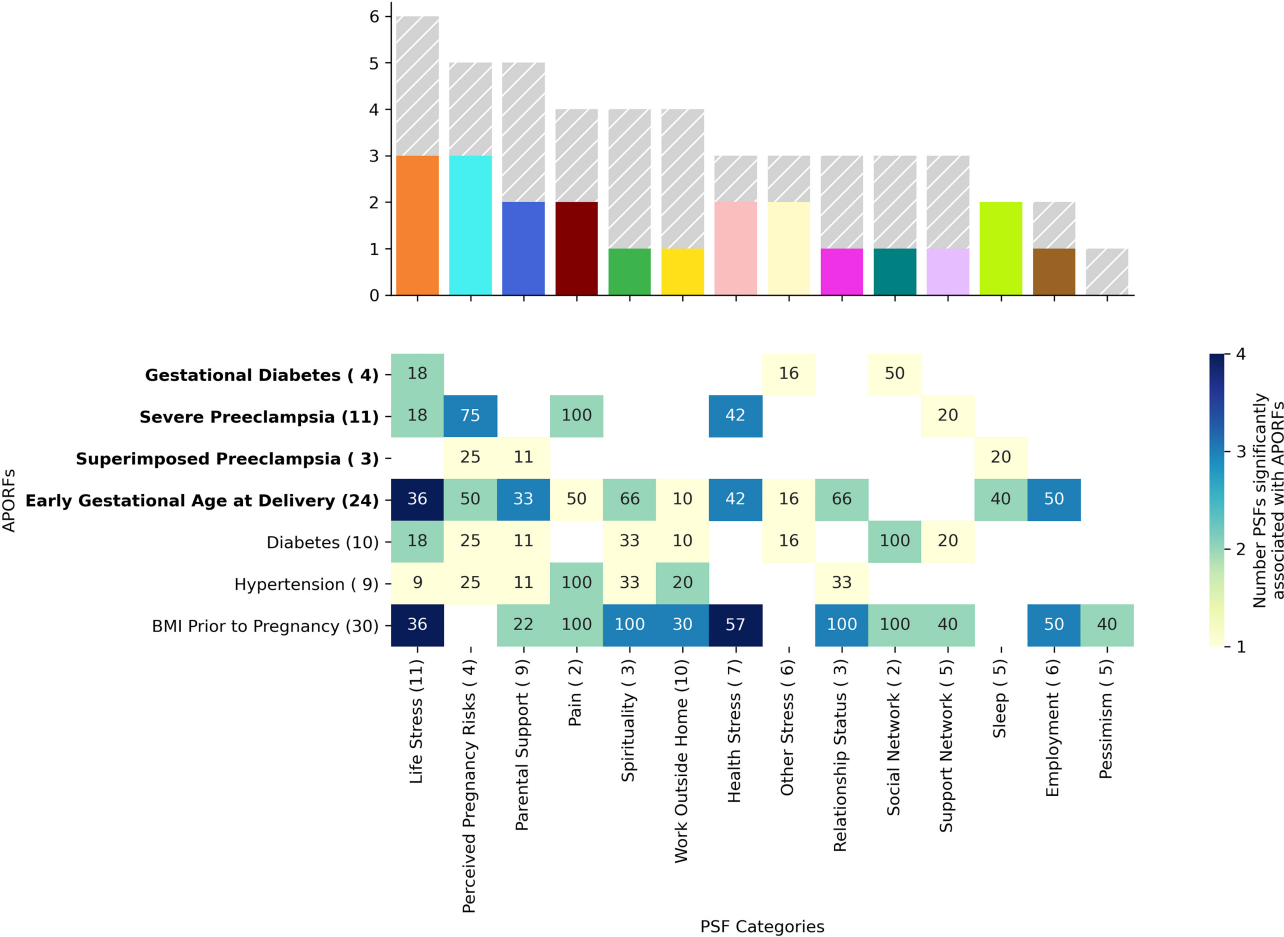
APORFs associated with categories of PSFs. The bar plot depicts the number of APORFs that are associated with each stress category (Supplementary Figure S4). Numbers adjacent to each APORF depict the number of categories significantly associated with each APORF. The height of the colored bar depicts the number of APOs associated with the PSF category, while the height of the gray bar depicts the number of RFs associated with the PSF category. The heatmap depicts the specific APORFs correlated with the PSF cluster, with darker colors representing a greater number of features from the cluster correlated with the outcome. The numbers in the heatmap represent the percentage of features from the cluster that was significant for the APORFs. The number of features in each cluster is stated in parentheses after each x-axis label. The life stress category was connected to the highest number of APORFs.

For the association of APORFs with PSFs in Figure 4, the Spearman's rho was calculated to determine correlation, and the corresponding *p*-value (*p* < 0.05) was used to determine significance. Only clusters that contained significantly predictive PSFs, as determined by *p*-value were included in Figure 4. To determine the most promising clusters for potential targeted interventions, the number of APORFs significantly associated with each PSF cluster was visualized (Figure 4). An APORF was considered to be significantly associated with a cluster if any PSF in the cluster was significantly associated with the APORF. A heatmap that further investigates the percentage of each PSF cluster correlated with APORFs was created (Figure 4). The percentages of significant individual PSFs normalized by the total PSFs in each category were visualized as numbers within the heatmap. The number of features per cluster is depicted in parentheses following PSF x-axis labels, and the number of significant categories related to each APORF is in parentheses following APORF y-axis labels.

**Figure 5:**
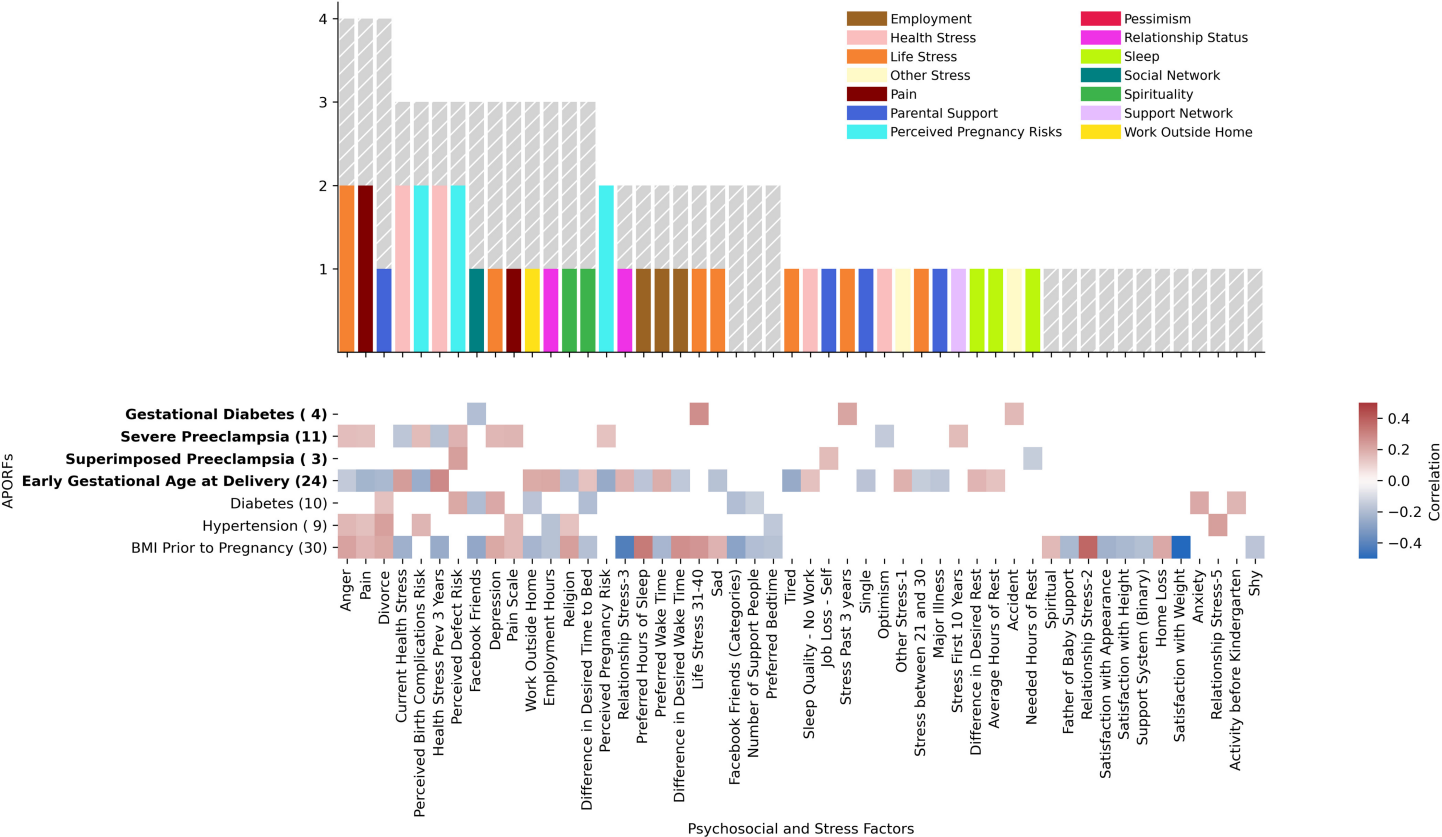
Number of APORFs significantly correlated with individual stress factors. The bar plot depicts PSFs, colored by cluster (cf. Supplementary Figure S4). Height of the bars represents the number of APORFs for which the PSF was significantly associated with APOs (colored bars) and RFs (gray bars). Hours of rest, why women thought they may experience birth defects (“perceived reason for defect”), and pain were correlated with the largest number of APORFs. The heatmap depicts Spearman correlation of PSFs with APORFs. Grey heatmap squares represent correlations of PSFs with APORFs that were not significant (significance was denoted by *p* < 0.05).

Next, we investigated the relationship between individual (within each cluster) PSFs and APORFs. Individual PSFs were significantly correlated with APORFs if *p* < 0.05 for Spearman correlations calculated. Individual PSFs, only included if they were significantly correlated with at least one APORF, were plotted based on the number of APORFs they were significantly correlated with (Figure 5). Individual PSFs, sorted based on the highest number of APORFs they were correlated with, were colored by their original clusters. Heatmaps depict the Spearman correlation value of each PSF with each APORF, with only PSFs with *p* < 0.05 included (Figure 5).

#### Methodological approach for linking stress to the immune system

**Figure 6:**
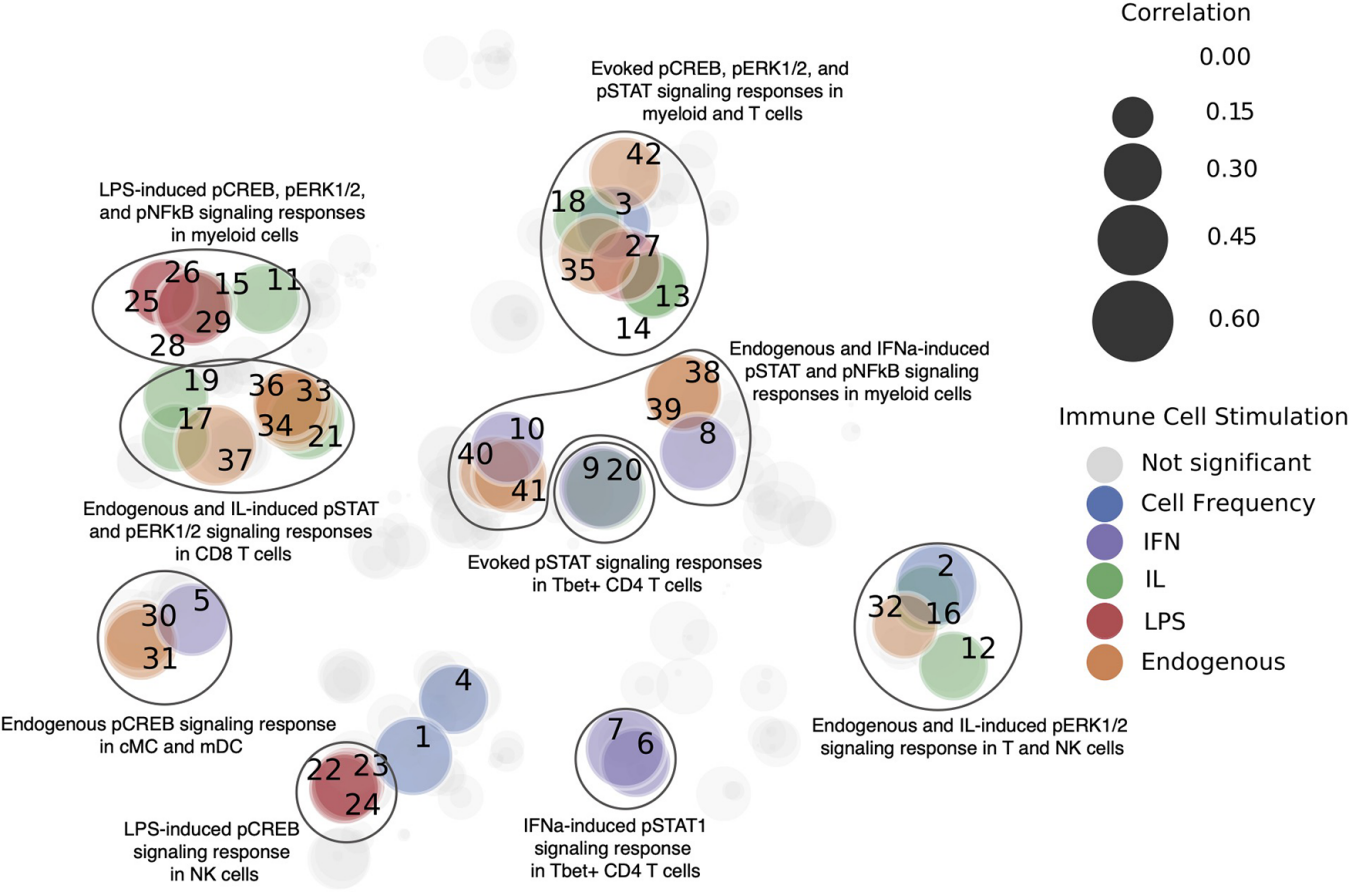
Immune features that are predictable from PSFs. Immune features were clustered by immune cell stimulation (designated by color), and models were trained to predict these features. Significant immune characteristics (*p* < 0.05 after correction *via* Benjamini-Hochberg false discovery rate) as predicted from the 79 original PSFs are colored. Prediction quality measured *via* Spearman correlation is represented by the size of the nodes. Each immune feature is labeled with a number, which maps to Supplementary Table S2. This analysis indicated that stress factors can predict components of the immune system, which paves the way for an integrative immune and stress model of pregnancy.

In a smaller subset of 14 patients who had participated in both studies, the same 79 features from the stress questionnaire from above were used to predict 534 single cell immune characteristics, i.e, measured single cells for each patient that were aggregated into cell frequencies and stimulus responses. A time point representation, a sequential integer from 1 through 4 representing the first, second, and third trimesters, and postpartum, was added to incorporate information about when immune cell measurements were taken. Ridge regression with grid search cross validation was used for prediction. The grid search was used to find the best alpha values (1, 0.1, 0.01, 0.001, 0.0001, 0) within each test fold. Feature values were scaled to unit variance. 10-fold cross validation was performed, for which a shuffled group K-fold was implemented to split patients for the test sets in order to keep data from the four time points for each patient within the same fold. The procedure was repeated 50 times. Spearman's rho was evaluated across all samples for each repetition, and a mean was taken for *p*-values and correlations for each immune feature. Benjamini-Hochberg False Discovery Rate Correction (alpha = 0.05) was then used on Spearman *p*-values. The results are visualized in Figure 6. Each node represents an immune feature. Their location is based on a two-dimensional embedding calculated by employing the t-distributed stochastic neighbor embedding (33) on the absolute values of the correlation vectors associated with each feature.

#### Prediction of adverse pregnancy outcomes from generated immune features

As only 14 patients in the cohort had data that included immune features, we generated immune features for the rest of 186 patients. These in-silico generated immune features were then used to predict APORFs. The 14 patients with immune system features were excluded from subsequent analyses to prevent bias. The immune features were generated by employing the coefficients from the ridge regression model used in the experiment for predicting immune features from PSFs. In particular, new coefficients were derived by taking the mean of the coefficients over all runs of the algorithm. Based on these coefficients a new linear model was used to predict immune cell characteristics from stress. For predicting APOs from the generated immune features, the same neural network architecture for the multitask and single task approaches in the prediction of APOs from stress was used; the generated immune features were the input into the neural network, and the output predicted was APOs.

## Results

### Distinct relations of adverse pregnancy outcomes

Analyses are based on our cohort of 200 pregnant women, 52 of whom experienced at least one APO during their pregnancy. Supplementary Figures S1, S2, and Supplementary Table S1 describe the demographics of the pregnant women, the distribution of BMI, and GA at delivery. Figure 2 represents the interconnectedness of APOs and RFs in our dataset based on Spearman correlation, confirming known relationships between APORFs established in prior studies (26, 34–37). There are biological underpinnings to these correlations; for instance, prior studies found that TGF-β is a common biological factor between preeclampsia and diabetes (38, 39). Diabetes was moderately correlated with hypertension and superimposed preeclampsia (40), and significantly correlated with every other APORF. Similarly, BMI prior to pregnancy was also significantly correlated with all outcomes (41). Finally, diabetes, hypertensions, superimposed and severed preeclampsia form a tightly connected cluster. These results highlight significant connections of APOs and RFs, suggesting that joint modeling may capture plausible underlying biological processes.

### Multitask machine learning improves prediction of adverse pregnancy outcomes

We applied MML to jointly model APOs together with RFs based on psychosocial and stress-related factors (PSFs). This not only leverages the previously mentioned interrelatedness of APORFs, but also increases the number of cases from which the model can learn. The latter supports the training process as ML models perform better with more balanced numbers of training samples for cases and controls. Case counts of the individual APOs and a comparison to the joint case count in a multitask setting are illustrated in Supplementary Figure S3. Consequently, multitask learning significantly improved the joint prediction of nearly all APOs compared to single task settings, which only consider one APO or RF at a time (Figure 3). Only for preeclampsia the model performance was consistent between the single and multitask approach. This further illustrates how multitask learning depends on the interrelatedness of the predicted outcomes, as preeclampsia was only marginally related to other APOs and RFs (cf. Figure 2). On the other hand, preeclampsia superimposed on preexisting hypertension (“Superimposed Pree”), which is strongly connected to the other modeled outcomes, benefited the most from the multitask approach. The ability of MML to improve prediction of APOs confirms their interconnectedness (∼50% were moderate or strong correlations). It also motivates the following study of stress factor categories that may help to improve the prevention of several APOs simultaneously.

### Stressor categories as well as individual stressors are related to multiple adverse pregnancy outcomes

Our previous analysis modeled the complex connections between an extensive set of PSFs and APORFs (see Supplementary Figure S4 for a visualization of significant PSFs related to each APORF). To understand this connection in more detail, we identify specific categories of PSFs and their connections to the studied APORFs using a clustering approach. Analogously to previous work (12), PSFs were clustered according to their correlation profiles (see Supplementary Figure S5 for a visualization of the categories). Categories such as perceived pregnancy risks, mental health (“life stress”), support network, and self-rated health (“health stress”), pain, and tiredness/fatigue (“sleep”) emerged as clusters. For example, the cluster related to “life stress” included: depression, happiness, anger, sadness, perceived loneliness, and stress in the past year. The clusters related to “perceived pregnancy risks” included perceived birth defects, perceived birth complications, and perceived pregnancy risk.

To understand the general association of these PSF categories with APORFs, we investigated how each PSF category and each individual PSF are associated with the studied APORFs (see Figures 4, 5, and Supplementary Figure S4). When quantifying the number of APORFs associated with any PSF in each PSF category (Figure 4), we observed that “life stress”, and “perceived pregnancy risks” categories were associated with most APOs (three out of four; see heatmap in Figure 4). These two categories were furthermore connected to several RFs (gray bars in Figure 4), with “life stress” covering all APORFs except superimposed preeclampsia on hypertension. Figure 4 shows a more detailed analysis regarding the number of PSFs in each category connected to APORFs.

We further investigated individual PSFs and their association with APORFs. For this, we visualized the number of APORFs significantly correlated with individual stressors (Figure 5). Here, when examining the individual PSFs in each of the PSF categories and their connection to APOs (see Figure 5, colored bars), PSFs from the “life stress” category that displayed significant correlations with APOs included depression and anger (associated with severe preeclampsia) as well as sadness and tiredness (associated with early gestational age at delivery). The cluster, “health stress,” included significant associations of lack of optimism with severe preeclampsia, as well as worries about health within the last three years (“health stress prev 3 years”) and stress around current health (“current health stress”) correlated with severe preeclampsia and gestational age at delivery. Specific “perceived pregnancy risks” correlated with APOs included worries about birth complications (“perceived birth complications risk”), defects (“perceived defect risk” and “perceived reason for defect”), and the pregnancy itself (“perceived pregnancy risk”) associated with one or two APOs each, including severe and superimposed preeclampsia and GA at delivery.

When investigating the detailed relationships between APOs and individual PSFs (see Figure 5), PSFs were frequently correlated with severe preeclampsia and GA at delivery. For example, pain, perceived birth complications risk, current health stress, and perceived pregnancy risk were significantly associated with both APOs simultaneously. Also, perceived defect risk co-occurred with severe and superimposed preeclampsia. These observations may point towards interventions that target the prevention of multiple APOs at a time. Along the same lines, interventions targeting life stressors as well as other PSF categories with PSFs not associated with several APOs simultaneously (see Figures 4, 5) can be promising targets, as their interrelated nature (Supplementary Figure S5) may provide a particularly large impact in the context of a targeted therapeutic intervention. Beyond the PSFs implicated by the cluster analysis above, PSFs like pain, divorce, or employment hours exhibited high numbers of relationships to APOs and RFs. While many APOs and RFs have strong connections to PSFs, individual connections with gestational diabetes and superimposed preeclampsia were limited.

Overall, these observations point to multiple, joint as well as individual PSFs, that are deeply intertwined with APORFs. For APOs, this focuses on severe preeclampsia as well as GA at delivery, with gestational diabetes and severe preeclampsia seemingly less connected with stress factors. These results introduce the potential for stress-based interventions targeting individual PSFs as well as PSF categories and suggest shared underlying biological pathways.

### Stress in early pregnancy impacts immune system signaling pathways

Although multifactorial, the biological mechanisms underlying observed associations between stressors and APOs likely implicate immunological factors. It is known that stress affects immune function (42), while multiple studies suggest that dysfunctional maternal immune adaptations to the developing fetus are critical to the pathogenesis of APOs (43–45). To investigate the relationship of the maternal immune system to PSFs, we employed a predictive modeling ML approach (single task). For a cohort of 200 pregnant women, PSFs were assessed during the first trimester while immune changes were captured for 14 pregnant women (a subset of the initial 200 pregnant women) over the course of pregnancy using a panel of antibodies for CyTOF. Models built using the PSF data predicted several immune features, including endogenous and stimulated signaling responses in both innate and adaptive immune cells (as measured by the abundance of phosphorylated intracellular signaling proteins representative for activated MyD88, Jak/STAT, mTOR, and NFkB pathways), and the relative abundance (frequency) of innate immune cells among all immune cells (Figure 6, Supplementary Table S2). The top 10 most predictable immune cell characteristics were dominated by activated signaling responses in CD4 + and CD8 + T cell populations; endogenous pERK in *γδ* T cells and native CD8+ T cells, and JAK/STAT signaling response to interleukins (IL-2, IL 6) or IFN*α* stimulation in naive Tbet + CD4 + and CD8 + naive T cells. Interestingly, mainly JAK/STAT (pSTAT1, pSTAT3, pSTAT5), MyD88 (pERK), and pCREB signaling responses in innate and adaptive immune cells were predicted from the PSFs, while only 6 out the 42 predictable characteristics (for which the Benjamini-Hochberg corrected *p*-value < 0.05) were part of the mTOR (prpS6) and NF*κ*B signaling pathway. In addition, PSFs were also able to predict the frequency of classical monocytes, CD16^neg^ NK cells, granulocytes, and naive Tbet + CD8 T cells. CREB can be activated (phosphorylated) downstream of a signaling pathway resulting from the binding of serotonin and noradrenaline to post-synaptic G-protein coupled receptors. Dysfunction of these neurotransmitters is implicated in major depressive disorder (46). Overall, we observe distinctive connections between PSFs and immune cell characteristics, which points towards the existence of an underlying biological pathway that may be directly connected to stress. This may for example enable the quantification of the effect of stress-related interventions to target APOs.

### A direct pathway connecting the stress, immune system, and adverse pregnancy outcomes triad

**Figure 7:**
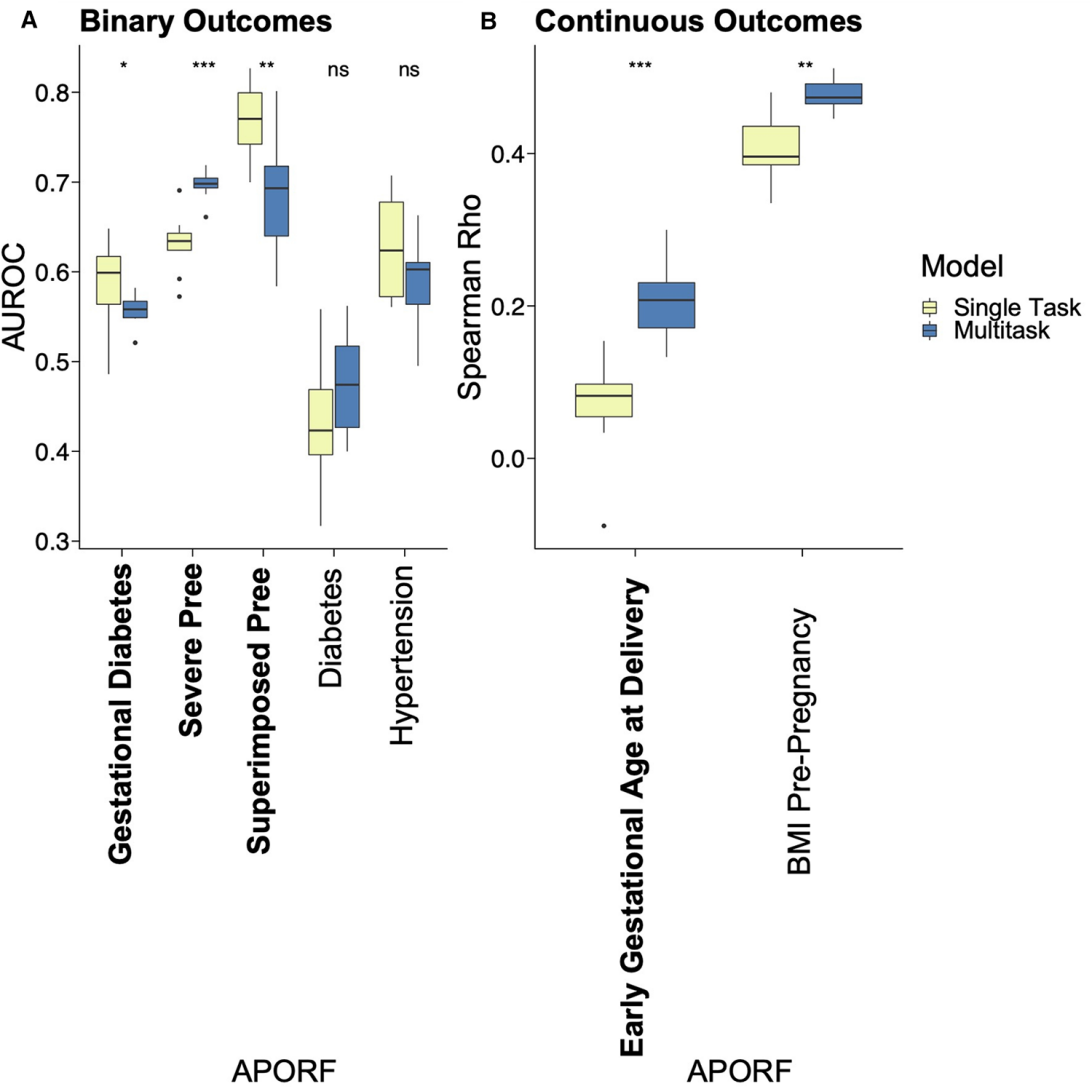
Multitask and single task machine learning analysis for the prediction of APOs based on generated immune features. APOs are bolded, and pathophysiological RFs are unbolded. Results of multitask models are blue, and single task models are yellow. Prediction of APOs was successful with generated immune features, confirming their biological relationship. Asterisks represent significant differences calculated by the Wilcoxon rank-sum test between single task and multitask approaches, with an increasing number of asterisks representing increased significance. Thresholds for significance include - ns: *p* > 0.05, *p <= 0.05, **p <= 0.01, ***p <= 0.001, and ****p <= 0.0001.

Because the previous experiment showed a connection between stress and immune system characteristics, we further investigated whether these biological signals were connected to APOs. As immune system data were available only for 14 healthy patients, we inferred the immune system state with our trained models for all patients at each timepoint in the remainder of our dataset, including patients with APOs. Here, we used the previously trained models, which successfully predicted a subset of immune system states from the original PSFs, and input PSFs for the remaining 186 women to in-silico generate their immune system states (Figure 6). Based on these generated immune system states, we applied single task as well as MML approaches analogous to our previous setup (multitask prediction of APOs) to successfully predict APOs from immune profiles (Figure 7). This not only confirms the connection of these biological factors to APOs but also points towards a direct pathway connecting stress, the immune system, and APOs.

## Discussion

### Impact of studying stress and adverse pregnancy outcomes

The relationship between stressors and APOs, along with the modifiable nature of stressors, introduces the possibility for actionable interventions. In prior studies, specific factors such as psychosocial stress, bereavement, and anxiety were correlated with individual APOs (11, 13, 47). Here, we aimed to simultaneously and comprehensively quantify the contribution of a multitude of stressors to understand those that contribute most to APOs. To this end, we used the extensive DQAQ-SPF questionnaire, which quantified psychosocial and stress factors and allowed individuals to rate the relative contributions of these stressors. In a previous study based on the same questionnaire (12), higher levels of deleterious factors were associated with PTB. These promising results, combined with the interrelatedness of APOs, suggested that a similar set of deleterious or protective factors could jointly affect multiple APOs simultaneously as reported in our study. The DQAQ-SPF allowed us to compare the impact of rarely studied stressors between and within categories of stressors (e.g., life stress, perceived pregnancy risks, health stress), as well as quantify their impact on each APO. Ultimately, this outlined approach could lay the foundation for developing targeted interventions based on readily accessible stress and biological parameters.

### Life stress, health concerns, and perceived pregnancy risks are implicated in adverse pregnancy outcomes in the studied population

The association of life stress with GA at delivery, severe preeclampsia, and gestational diabetes, and the correlation of worries about health (“health stress”) with preeclampsia and GA at delivery, as found in the studied population, may point towards specific psychometric targets to alleviate, or prevent APOs in a joint manner. Additionally, the observed high correlation of APOs with health concerns and perceived pregnancy risks highlights the impact that negative perception can have on the health of the mother and fetus. This may enable preventive approaches such as in-depth counseling by providers in addition to pharmacological treatments to clarify and assuage these worries. Support and social networks were both correlated with GA at delivery. These results are consistent with current literature, as life stress, including feelings of guilt, loss of control, and fear, as well as pregnancy perception, body image, marital status, and work have been correlated with pregnancy complications or loss (48, 49). Support and social networks have additionally been shown as crucial to successful management of chronic disease (50). Prior studies corroborated that life stress, health stress, and perceived pregnancy risks were related to APOs, lending credence to the benefits of targeting implicated stressors.

### Successful joint modeling of adverse pregnancy outcomes emphasizes their relatedness

One of the main contributions of our work is to model multiple APOs simultaneously to better capture their biological interconnectedness. Although APOs are comparably rare, and thus challenging to model, their biological relationship and frequent presence as comorbidities provide an avenue for joint modeling approaches like MML to increase predictive power. As mentioned in the introduction, hypertension during pregnancy is correlated with diabetes and BMI; gestational diabetes is connected to hypertensive disorders, preeclampsia, and PTB; and prior preeclampsia is tied to an increased risk of hypertension and diabetes (26, 27, 34). As shown by our results, MML successfully models APOs simultaneously, outperforming single task approaches. While the overall performance metrics still need to be improved for practical applications, the superior performance of MML emphasizes the interconnectedness of APOs and illustrates the potential of building on joint combinatorial outcomes to significantly increase predictive power. This motivates further research in the joint study of the corresponding biological systems in a holistic manner and may lead to an integrated and actionable understanding of APOs.

### The joint pathway of stress factors, immune system, and adverse pregnancy outcomes

Decades of research have shown that a pregnancy success relies on finely orchestrated immunological cross-talk between the mother and the fetus, with pregnancy complications being associated with feto-maternal immune dysfunction (43–45). In addition, exposing mice to stress has been shown to suppress their immune system (51), while immune dysregulation could also be observed in humans under chronic stress exposure (42). This connection between the immune system, stress, and APOs introduces the possibility of implementing targetable and measurable stress-based interventions. In the study presented here, stressors were able to predict several immune features in a cohort of pregnant women suggesting a potential link between stress and APOs through immune system factors. Interestingly, pSTAT5 signaling responses in Tbet + CD4 T cells were predictable from stressors, and the immune model used was successfully able to predict adverse pregnancy outcomes, which corroborates prior work that has shown pSTAT5 signaling in Tbet + CD4 T cells can classify preeclamptic pregnancies from controls (43), and pSTAT5 signal in CD4 T cells can predict GA (32). Overall, the predicted features spanned innate and adaptive immune populations and their major signaling cascades, suggesting common neuroendocrine factors which affect immunity *via* globally expressed receptors, and thus mediate an immune-system-wide stress response. This general stress adaptation might be most efficiently therapeutically targeted by intervening with the stressors itself. Such interventions could include mindfulness-based stress reduction protocols, which have shown promise in reducing stress in pregnant (52, 53) and non-pregnant women (54), with a recent meta-analysis demonstrating their beneficial post-interventional impact on immunity-related biomarkers in non-pregnant participants (55). Pregnancy-adapted protocols continue to be subject of clinical trials (56, 57).

Moreover, to investigate the potential for connections among stress, the immune system, and APOs, stressors were used to in-silico generate immune data for a part of our patient cohort. These generated immune features successfully predicted not only GA, but other APOs as well, confirming the relationship between stressors, immunity, and APOs (Figure 7). This shows that a model for predicting immune states from stress profiles (PSFs) that is solely trained on “healthy” pregnancies can still capture immune state characteristics of abnormal pregnancies (related to APOs). This indicates that pregnancies and particularly their immune profiles are on a spectrum (or likely, multiple spectrums) in contrast to belonging to a discrete class like “healthy” and “abnormal”. Overall, this points towards a joint pathway and may enable possibilities for the assessment of stress-based interventions to improve APOs.

### Stress-related therapeutics for adverse pregnancy outcomes

The predictive stressors elucidated in this study may provide the foundation for further studies to devise treatments that improve the outcome of multiple APOs simultaneously. The observed connection of life stressors and APOs may introduce possibilities for interventions that can be combined with pharmaceuticals for the best outcomes. This may have particularly strong impact on APOs that are difficult to treat, like preeclampsia (10). For example, though in some cases medications are indicated to treat symptoms of preeclampsia (10), currently the most effective treatment is delivery. However, delivery may result in PTB, which is correlated with negative health outcomes for the mother and the fetus. Therefore, treatments based on targeting stress may have the potential for effective noninvasive interventions.

The potential for noninvasive therapies that target stressors implicated in this study is tremendous. Randomized trials have shown that mind-body techniques and other nonpharmaceutical interventions including tai chi, guided imagery, progressive muscle relaxation, and yoga, have reduced mood symptoms during pregnancy (58). Noninvasive techniques such as massage during pregnancy and postpartum have also been valuable in improving mood, decreasing perception of pain during labor (59). In addition, the strong correlation of perception with APOs may point towards the possibility that altering perception could improve outcomes. As accumulation of evidence improves perception, reassurance from care teams could prove helpful (60). Additionally, further education has improved self-efficacy and decreased severity of illness perceptions in conditions such as type II diabetes, and similar techniques could be applied in women at high risk from pregnancy complications (61). Such techniques may further synergize with pharmaceuticals to target significantly implicated stressors to decrease the risk of multiple APOs.

### Study limitations

The results in our study point towards a joint pathway connecting stressors, biology, and APOs, that may allow for preventing APOs by targeting stressors and measuring progress through biological assays. In this context, our MML model as well as the generative approach for deriving immune system measurements, compensates for relatively small numbers of APOs. However, particularly for predicting APOs from immune system features the severely limited training data (regarding sample size as well as its focus on healthy samples) significantly reduced the advantages of the MML approach. Additionally, while building models on generated data in this study, and employing a rigorous repeated cross-validation scheme, allowed for first insights into the connection between multiple APOs, the immune system, and PSFs, further research requires explicitly measured immune features to validate the observations from our analysis. Thus, in future work, additional data, especially with respect to biological measurements and more APO samples, will help strengthen our findings. In addition, self-reported data generally introduces bias, as participants may alter answers based on experiences or judgment (62) and will need further investigation in the context of our study particularly with regards to background information available to the patient; for instance, the amount of information a patient has about their medical history may vary. Extended studies with larger cohorts and specifically designed questions may enable investigating the causal structure of the observed effects and account for the confounding factors inherent to self-assessed stress related questions, particularly in the context of the complex interconnected nature of APOs and RFs.

## Conclusion

Elucidating connections among stress, multiple APOs simultaneously, and immune characteristics using ML-based models are initial steps to facilitate the implementation of individualized, integrative models of pregnancy in clinical decision making, with a particular focus on the diagnosis and prevention of APOs. The success of the employed multitask approach confirms the interrelatedness of APOs and the potential for interventions that simultaneously target multiple APOs. Furthermore, the connection between stress and the immune system, where generated immune features were also correlated with APOs, elucidates an underlying biological link. While our study only represents a first foray into this promising area of research, the wide applicability of identifying impactful stressors with questionnaires and their modifiable nature may ultimately allow for accessible interventions, with success potentially measured biologically through immune characteristics.

## Data Availability

Code and data for reproduction of results in this paper can be downloaded from the following link: https://nalab.stanford.edu/pregnancy-stress-multitask/. Stress related questions (except for birth “complications” and “defects”) are from the DQAQ-SPF Questionnaire, copyright (2020) Firdaus S. Dhabhar and the University of Miami, jointly with Stanford University. All rights reserved. This questionnaire may not be used fully or partially, reproduced, displayed, modified, or distributed without the express prior written permission from Dr. Dhabhar (dhabhar@gmail.com). Further inquiries can be directed to the corresponding author.
