# Supplementary material for: Revealing the impact of lifestyle stressors on the risk of adverse pregnancy outcomes with multitask machine learning
Source: Front Pediatr. 2022 Dec 13;10:933266. doi: 10.3389/fped.2022.933266 (PMC9793100; doi:10.3389/fped.2022.933266)
Supplement: Supplementary file 1 [file Datasheet1.pdf]

Supplementary Material

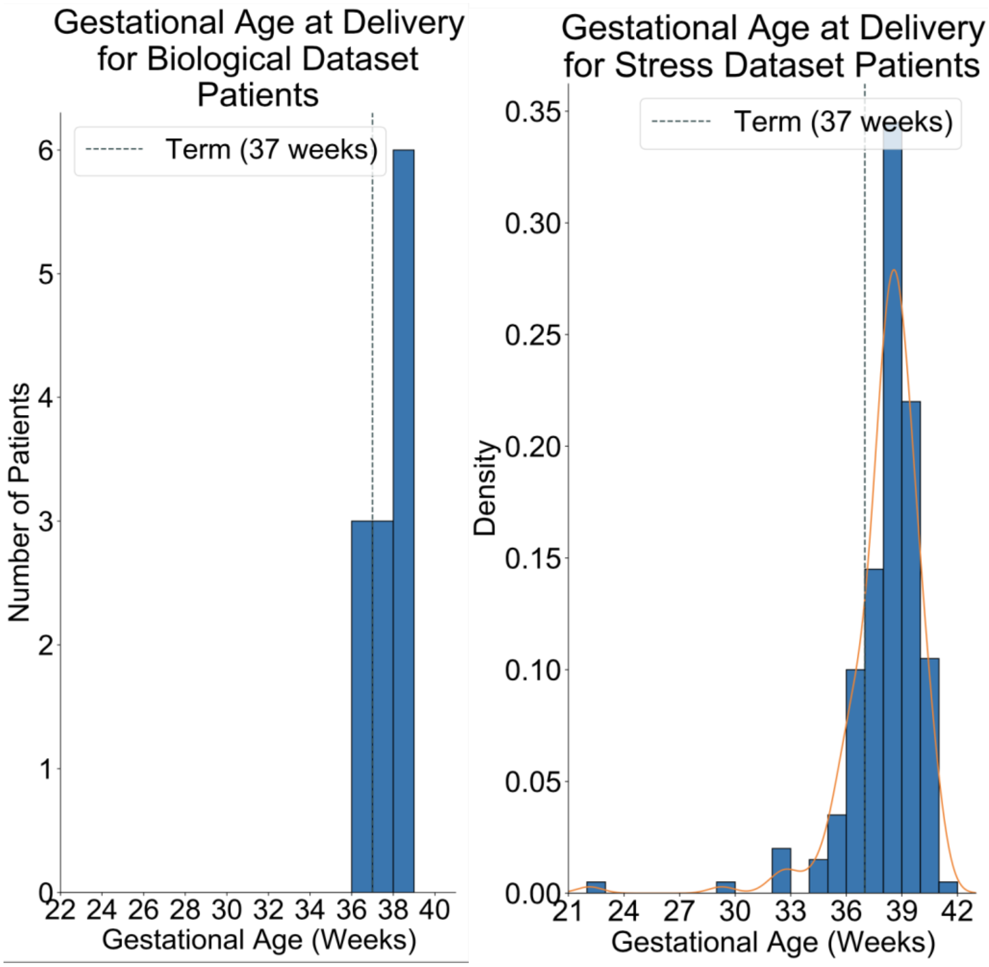

Supplementary Figure S1. *Distribution of gestational age (GA) at delivery for stress and biological dataset patients.*

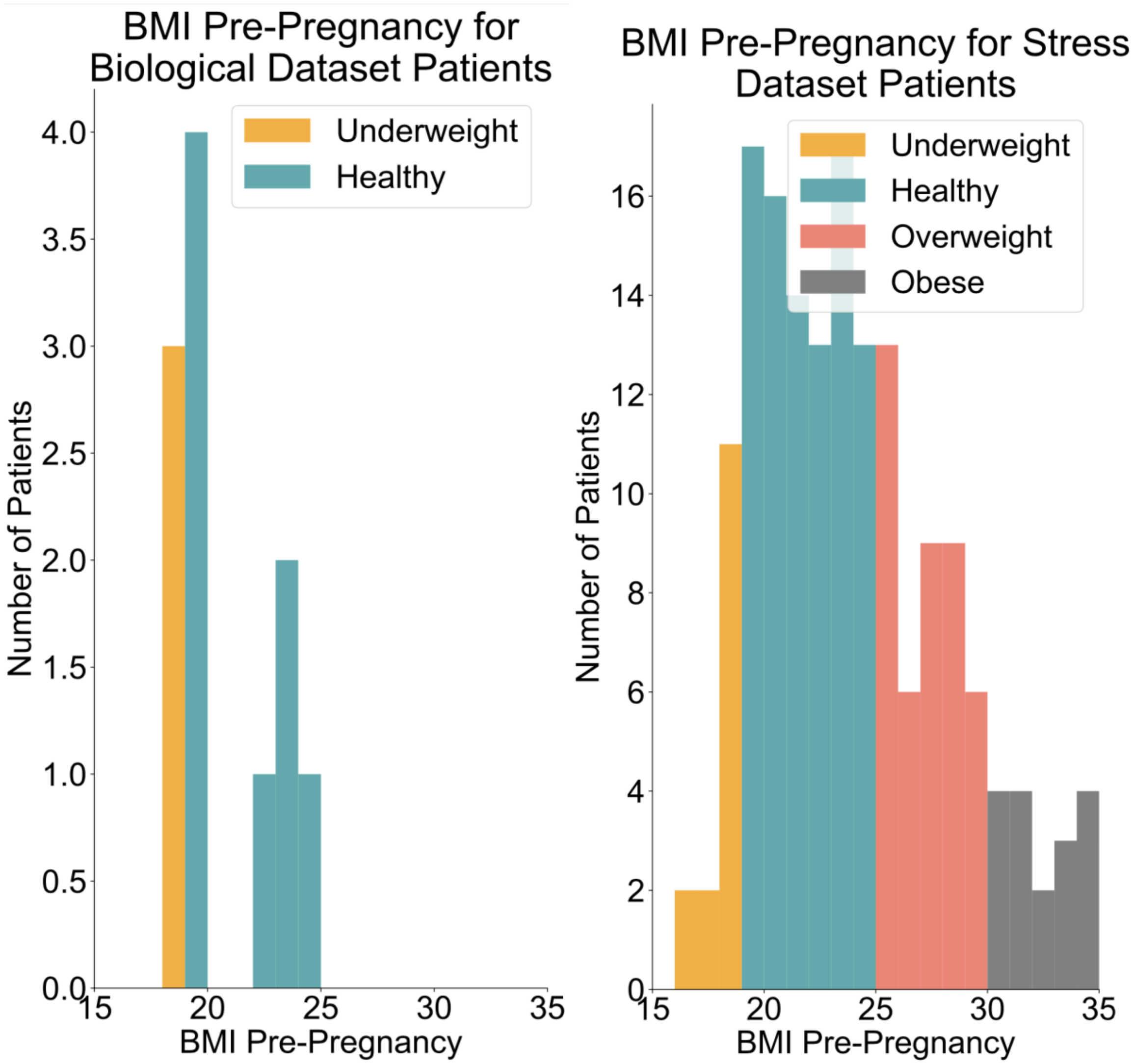

Supplementary Figure S2. Distribution of body mass index (BMI) pre-pregnancy for stress and biological dataset patients.

Supplementary Table 1.1. *Demographic information for outcomes (superimposed preeclampsia).*

	Superimposed (n=6)	Not Superimposed (n=194)
**Race**		
**White**	50.00%	56.35%
**Hispanic**	16.67%	13.07%
**Asian-Chinese**	0.00%	5.00%
**Black**	16.67%	4.52%
**Filipino**	0.00%	5.00%
**Indian**	0.00%	5.00%
**Asian-Vietnamese**	0.00%	2.00%
**Unknown**	16.67%	1.51%
**Asian-Unspecified**	0.00%	1.50%
**Decline**	0.00%	1.00%
**Asian-Korean**	0.00%	1.00%
**Asian-Japanese**	0.00%	1.00%
**Asian-Thai**	0.00%	0.50%
**Pacific Islander**	0.00%	0.50%
**Maternal Age**		
**≥30**	83.33%	67.18%
**<30**	16.67%	31.66%
**Maternal Education**		
**Post-undergraduate degree**	0.00%	37.00%
**Bachelors**	16.67%	23.12%
**Some college**	33.33%	17.17%
**Other**	0.00%	9.00%
**Some high school**	33.33%	5.56%
**High school diploma or equivalent**	0.00%	5.00%
**No high school**	16.67%	0.50%
**BMI**		
**Normal**	16.67%	53.04%
**Overweight**	33.33%	22.78%

**Obese**	50.00%	17.32%
**Underweight**	0.00%	4.40%
**Marital Status**		
**Married**	33.33%	72.96%
**Living with partner**	33.33%	13.27%
**Single**	16.67%	9.14%
**Decline**	16.67%	2.03%
**Divorced**	0.00%	0.51%
**Gestational Age at Delivery (Weeks)**		
**37+**	33.33%	92.35%
**32-36**	50.00%	6.15%
**<32**	16.67%	0.51%
**Total**	6	194

Supplementary Table 1.2. *Demographic information for outcomes (severe preeclampsia).*

	Severe Preeclampsia (n=13)	No Preeclampsia (n=189)
**Race**		
**White**	46.15%	55.67%
**Hispanic**	7.69%	13.07%
**Asian-Chinese**	0.00%	5.00%
**Black**	7.69%	4.52%
**Filipino**	0.00%	5.00%
**Indian**	7.69%	4.52%
**Asian-Vietnamese**	7.69%	1.51%
**Unknown**	7.69%	1.51%
**Asian-Unspecified**	15.38%	0.51%
**Decline**	0.00%	1.00%
**Asian-Korean**	0.00%	1.00%
**Asian-Japanese**	0.00%	1.00%
**Asian-Thai**	0.00%	0.50%
**Pacific Islander**	0.00%	0.50%
**Maternal Age**		
**≥30**	69.23%	66.49%
**<30**	30.77%	30.61%
**Maternal Education**		
**Post-undergraduate degree**	15.38%	36.36%
**Bachelors**	23.08%	22.34%
**Some college**	23.08%	16.75%
**Other**	15.38%	8.08%
**Some high school**	15.38%	5.56%
**High school diploma or equivalent**	0.00%	5.00%
**No high school**	7.69%	0.50%
**BMI**		
**Normal**	30.77%	52.25%
**Overweight**	30.77%	21.91%

**Obese**	38.46%	16.38%
**Underweight**	0.00%	4.40%
**Marital Status**		
**Married**	61.54%	72.11%
**Living with partner**	15.38%	13.27%
**Single**	15.38%	8.67%
**Decline**	7.69%	2.03%
**Divorced**	0.00%	0.51%
**Gestational Age at Delivery (Weeks)**		
**37+**	53.85%	92.15%
**32-36**	38.46%	5.18%
**<32**	7.69%	0.51%
**Total**	13	187

Supplementary Table 1.3. *Demographic information for outcomes (hypertension).*

	Hypertension (n=18)	No Hypertension (n=182)
**Race**		
White	38.89%	55.44%
Hispanic	27.78%	11.28%
Asian-Chinese	5.56%	4.52%
Black	16.67%	3.55%
Filipino	5.56%	4.52%
Indian	0.00%	5.00%
Asian-Vietnamese	0.00%	2.00%
Unknown	5.56%	1.51%
Asian-Unspecified	0.00%	1.50%
Decline	0.00%	1.00%
Asian-Korean	0.00%	1.00%
Asian-Japanese	0.00%	1.00%
Asian-Thai	0.00%	0.50%
Pacific Islander	0.00%	0.50%
**Maternal Age**		
≥30	77.78%	65.59%
<30	22.22%	30.61%
**Maternal Education**		
Post-undergraduate degree	11.11%	36.36%
Bachelors	22.22%	21.94%
Some college	27.78%	15.90%
Other	0.00%	9.00%
Some high school	22.22%	4.59%
High school diploma or equivalent	11.11%	4.04%
No high school	5.56%	0.50%
**BMI**		
Normal	27.78%	51.98%
Overweight	33.33%	21.02%

**Obese**	38.89%	15.43%
**Underweight**	0.00%	4.40%
**Marital Status**		
**Married**	52.94%	71.96%
**Living with partner**	23.53%	12.37%
**Single**	11.76%	8.67%
**Decline**	11.76%	1.53%
**Divorced**	0.00%	0.51%
**Gestational Age at Delivery (Weeks)**		
**37+**	66.67%	91.94%
**32-36**	27.78%	5.18%
**<32**	5.56%	0.51%
**Total**	18	182

Supplementary Table 1.4. *Demographic information for outcomes (diabetes).*

	Diabetes (n=11)	No Diabetes (n=189)
**Race**		
**White**	36.36%	56.12%
**Hispanic**	18.18%	12.63%
**Asian-Chinese**	9.09%	4.52%
**Black**	0.00%	5.00%
**Filipino**	0.00%	5.00%
**Indian**	9.09%	4.52%
**Asian-Vietnamese**	0.00%	2.00%
**Unknown**	27.27%	0.51%
**Asian-Unspecified**	0.00%	1.50%
**Decline**	0.00%	1.00%
**Asian-Korean**	0.00%	1.00%
**Asian-Japanese**	0.00%	1.00%
**Asian-Thai**	0.00%	0.50%
**Pacific Islander**	0.00%	0.50%
**Maternal Age**		
**≥30**	81.82%	66.49%
**<30**	18.18%	31.31%
**Maternal Education**		
**Post-undergraduate degree**	18.18%	36.36%
**Bachelors**	0.00%	23.50%
**Some college**	9.09%	17.59%
**Other**	0.00%	9.00%
**Some high school**	36.36%	4.59%
**High school diploma or equivalent**	18.18%	4.04%
**No high school**	18.18%	0.00%
**BMI**		
**Normal**	9.09%	53.04%
**Overweight**	36.36%	21.91%

**Obese**	54.55%	15.91%
**Underweight**	0.00%	4.40%
**Marital Status**		
**Married**	45.45%	71.79%
**Living with partner**	36.36%	12.24%
**Single**	9.09%	9.05%
**Decline**	9.09%	2.01%
**Divorced**	0.00%	0.50%
**Gestational Age at Delivery (Weeks)**		
**37+**	45.45%	91.28%
**32-36**	45.45%	5.13%
**<32**	9.09%	0.50%
**Total**	11	189

Supplementary Table 1.5. *Demographic information for outcomes (gestational diabetes).*

	Gestational Diabetes (n=29)	No Gestational Diabetes (n=171)
**Race**		
**White**	48.28%	53.76%
**Hispanic**	17.24%	11.28%
**Asian-Chinese**	13.79%	3.06%
**Black**	3.45%	4.52%
**Filipino**	3.45%	4.52%
**Indian**	0.00%	5.00%
**Asian-Vietnamese**	6.90%	1.01%
**Unknown**	6.90%	1.01%
**Asian-Unspecified**	0.00%	1.50%
**Decline**	0.00%	1.00%
**Asian-Korean**	0.00%	1.00%
**Asian-Japanese**	0.00%	1.00%
**Asian-Thai**	0.00%	0.50%
**Pacific Islander**	0.00%	0.50%
**Maternal Age**		
**≥30**	86.21%	63.43%
**<30**	13.79%	30.61%
**Maternal Education**		
**Post-undergraduate degree**	33.33%	33.68%
**Bachelors**	16.67%	21.54%
**Some college**	20.00%	15.46%
**Other**	6.67%	8.08%
**Some high school**	10.00%	5.08%
**High school diploma or equivalent**	10.00%	3.55%
**No high school**	3.33%	0.50%
**BMI**		
**Normal**	28.00%	51.43%
**Overweight**	32.00%	20.11%

**Obese**	40.00%	13.95%
**Underweight**	4.00%	3.87%
**Marital Status**		
**Married**	64.29%	70.56%
**Living with partner**	28.57%	10.53%
**Single**	3.57%	9.14%
**Decline**	3.57%	2.03%
**Divorced**	0.00%	0.51%
**Gestational Age at Delivery (Weeks)**		
**37+**	86.21%	91.33%
**32-36**	10.34%	6.15%
**<32**	3.45%	0.51%
**Total**	29	171

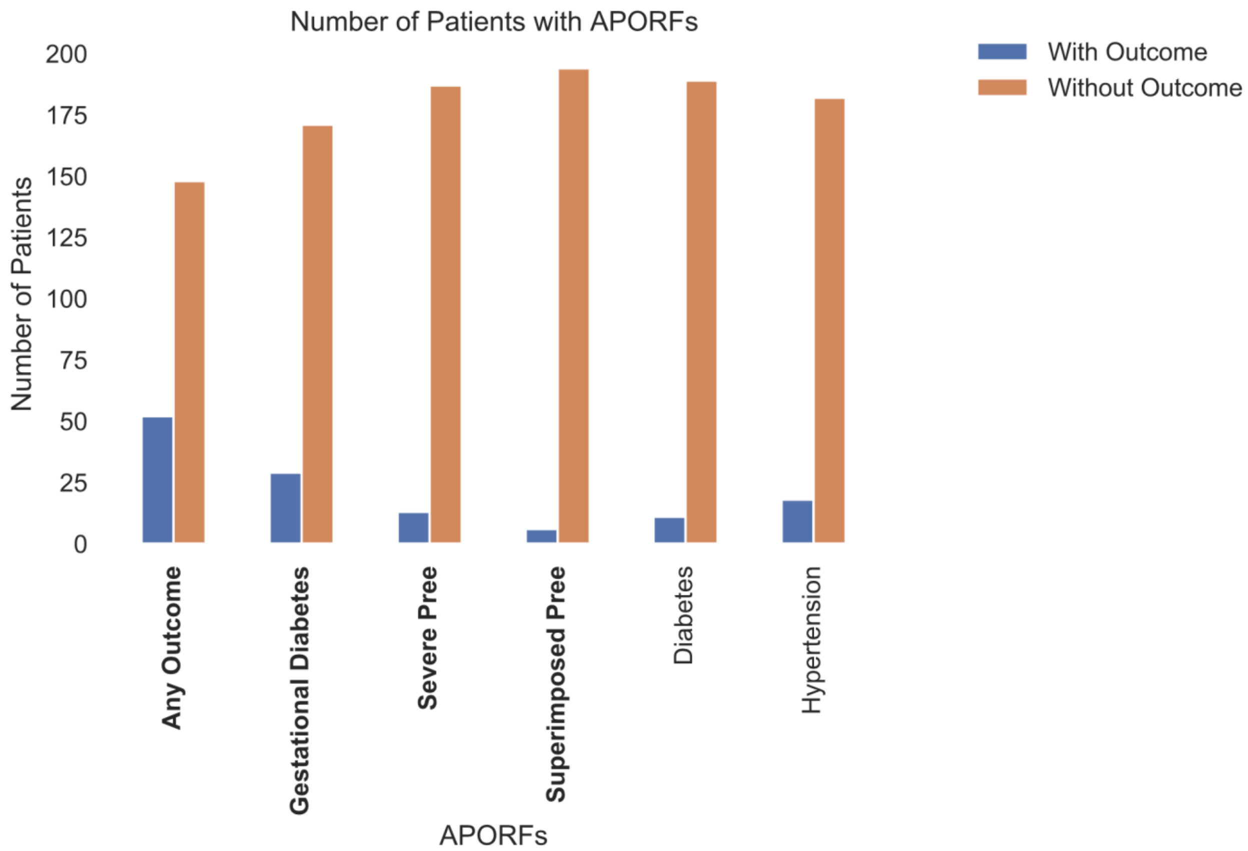

Supplementary Figure S3. *Number of patients who experienced any of the adverse pregnancy outcomes (APOs) or risk factors (RFs) and number of patients for each of the APOs or RFs individually. "Pree" is an abbreviation for preeclampsia.*

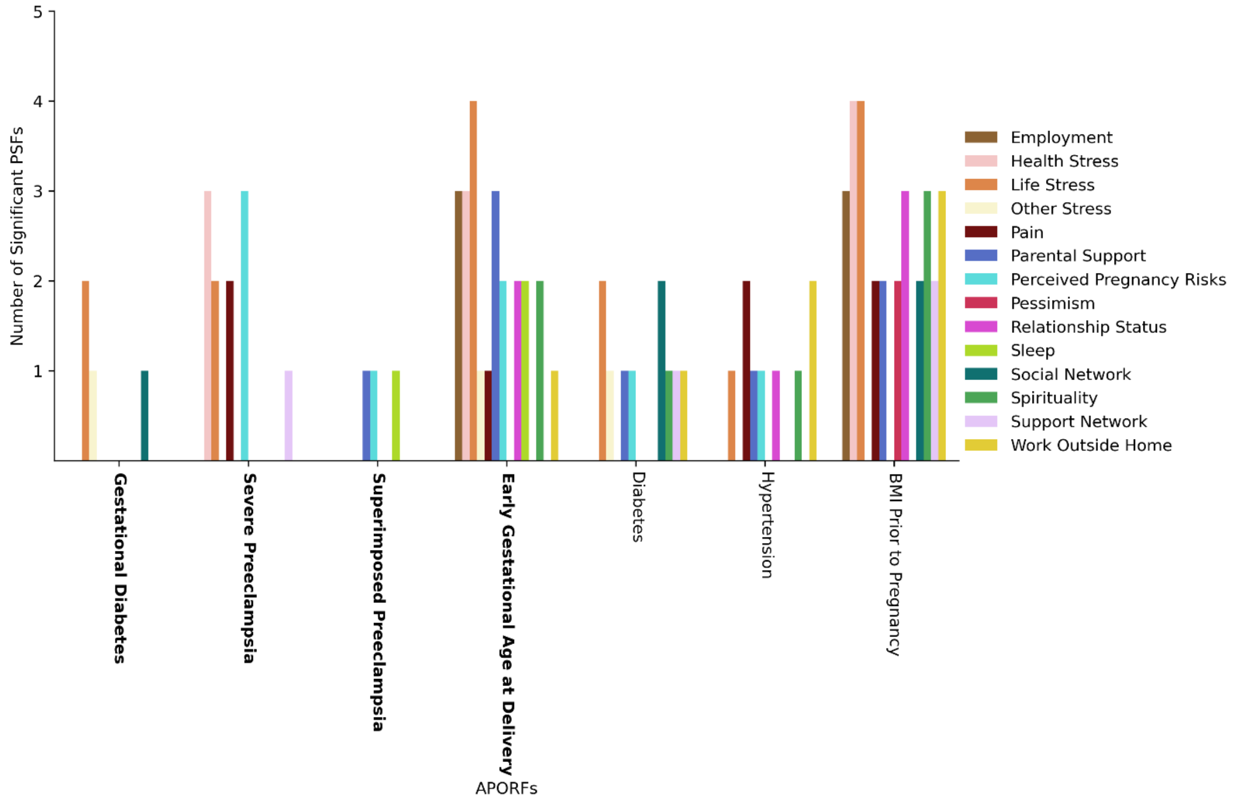

Supplementary Figure S4: Number of significant stressors colored by stress category for each adverse pregnancy outcome (APO). *Life stress, mental health, concern regarding health, and stress from pain were top predictive categories. There was significant overlap in terms of the stress categories predictive of APOs (similar colors seen amongst APOs), emphasizing their commonalities.*

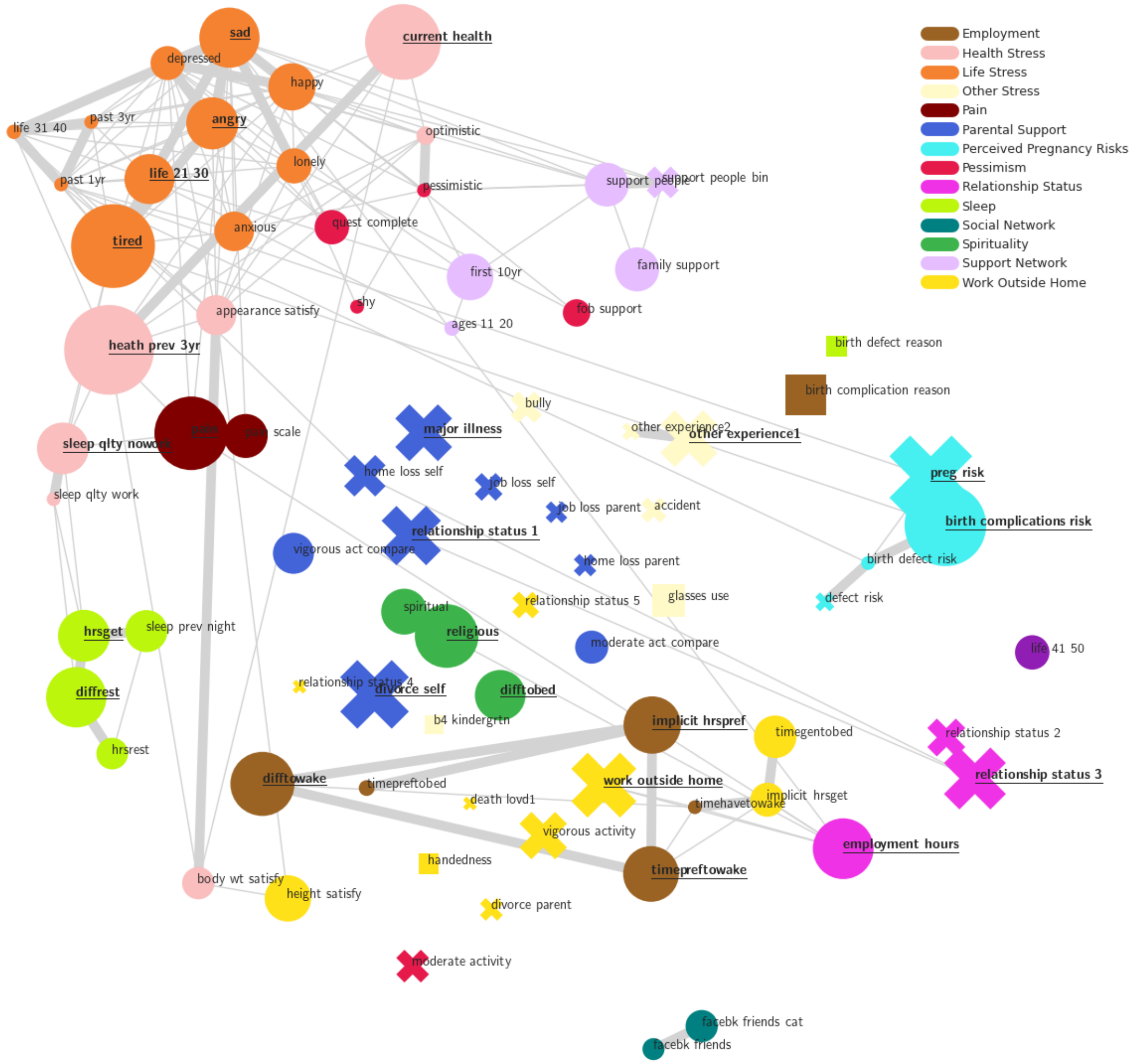

Supplementary Figure S5: Interdependency network of stress features and the strength of their association with early gestational age (GA) at delivery. *Each node corresponds to a feature derived from the Dhabhar Quick-Assessment Questionnaire for Stress and Psychosocial Factors^{TM} (DQAQ-SPF^{TM}). Clusters are based on analysis done in Becker et al., 2021***.** *The closer the features, the more similar they can be considered with regard to their correlation structure. The edges represent strong correlation between features that pass a Bonferroni corrected p-value threshold of <0.05. Thin edges represent absolute Spearman correlations of >0.3 and thick edges represent absolute correlations >0.5. As an illustration for a single APORF, node sizes represent the strength of association between GA (days) and the corresponding feature based on the p-value of the Spearman correlation. The colors represent clusters of closely related features. Shape of the node represents the type of variable (circle=continuous, cross=binary, square=categorical).*

Supplementary Table 2: *Spearman correlations and FDR-bh corrected p-values for immune cell characteristics predictable from stress, ranked according to p-value. IFNa at 100 ng/mL. LPS100; IL100; IL-2, IL-6 at 100 ng/mL each. IFNa100; LPS at 100 ng/mL*

Immune Cell	Index	Correlation	pvalue
**CD56+CD16-NKcells**	1	5.74E-01	4.20E-05
**cMCs**	2	6.13E-01	2.97E-06
**Gr**	3	5.03E-01	9.49E-05
**Tbet+CD8+Tcells naive**	4	4.53E-01	9.21E-04
**intMCs STAT1 IFNa100**	5	4.97E-01	2.59E-04
**Tbet+CD4+Tcells mem STAT1 IFNa100**	6	4.45E-01	3.65E-03
**Tbet+CD4+Tcells naive STAT1 IFNa100**	7	5.93E-01	5.36E-05
**intMCs STAT5 IFNa100**	8	5.48E-01	2.52E-05
**Tbet+CD4+Tcells naive STAT5 IFNa100**	9	6.47E-01	2.74E-04
**TCRgd+Tcells STAT5 IFNa100**	10	4.98E-01	1.63E-04
**CD8+Tcells mem ERK IL100**	11	4.65E-01	4.41E-04
**CD8+Tcells ERK IL100**	12	4.49E-01	9.01E-04
**cMCs ERK IL100**	13	4.30E-01	1.20E-03
**M-MDSC ERK IL100**	14	4.15E-01	1.83E-03
**mDCs ERK IL100**	15	4.04E-01	3.05E-03
**Tbet+CD4+Tcells mem ERK IL100**	16	4.28E-01	2.93E-03
**CD8+Tcells STAT1 IL100**	17	4.30E-01	2.51E-03
**CD8+Tcells mem STAT3 IL100**	18	4.36E-01	1.67E-03
**Gr STAT3 IL100**	19	4.02E-01	3.19E-03
**Tbet+CD4+Tcells naive STAT3 IL100**	20	6.25E-01	1.95E-06
**Tbet+CD8+Tcells naive STAT3 IL100**	21	5.48E-01	2.89E-05

**CD16+CD56-NKcells CREB LPS100**	22	4.41E-01	7.59E-04
**CD56+CD16-NKcells CREB LPS100**	23	4.87E-01	1.75E-04
**CD7+NKcells CREB LPS100**	24	4.79E-01	2.08E-04
**cMCs CREB LPS100**	25	4.03E-01	2.41E-03
**M-MDSC CREB LPS100**	26	4.23E-01	1.37E-03
**mDCs CREB LPS100**	27	4.90E-01	1.60E-04
**cMCs NFkB LPS100**	28	5.14E-01	8.50E-05
**M-MDSC NFkB LPS100**	29	5.50E-01	9.31E-05
**cMCs CREB Unstim**	30	4.66E-01	3.80E-04
**mDCs CREB Unstim**	31	5.00E-01	1.87E-04
**CD16+CD56-NKcells ERK Unstim**	32	4.01E-01	3.51E-03
**CD8+Tcells naive ERK Unstim**	33	6.28E-01	1.34E-05
**CD8+Tcells ERK Unstim**	34	5.36E-01	7.87E-05
**Tbet+CD4+Tcells naive ERK Unstim**	35	5.80E-01	3.63E-04
**Tbet+CD8+Tcells naive ERK Unstim**	36	4.71E-01	2.27E-03
**TCRgd+Tcells ERK Unstim**	37	6.15E-01	1.09E-06
**cMCs NFkB Unstim**	38	5.45E-01	6.17E-05
**M-MDSC NFkB Unstim**	39	5.45E-01	3.60E-04
**mDCs S6 Unstim**	40	4.89E-01	4.67E-04
**M-MDSC STAT5 Unstim**	41	5.14E-01	2.09E-04
**Tbet+CD4+Tcells naive STAT5 Unstim**	42	4.81E-01	3.24E-03

Supplementary Table 3: *Significant stress features mapped from original feature names to readable format.*

Readable format	Feature name	HTML color code (PSF category)
Perceived Reason for Defect	birth_defect_reason	#bcf60c
Pain	stress_pain	#800000
Hours of Rest	hrsrest	#bcf60c
Divorce	stress_divorce_self	#4363d8
Difference in Desired Time to Bed	difftobed	#3cb44b
Perceived Defect Risk	defect_risk	#46f0f0
Perceived Birth Complications Risk	birth_complications_risk	#46f0f0
Optimism	stress_optimistic	#fabebe
Health Stress Prev 3 Years	stress_heath_prev_3yr	#fabebe
Current Health Stress	stress_current_health	#fabebe
Religion	stress_religious	#3cb44b
Pain Scale	stress_pain_scale	#800000
Depression	stress_depressed	#f58231
Anger	stress_angry	#f58231
Perceived Pregnancy Risk	preg_risk	#46f0f0
Relationship Stress-3	stress_relationship_status___3	#f032e6
Relationship Stress-2	stress_relationship_status___2	#f032e6
Life Stress 31-40	stress_life_31_40	#f58231
Home Loss	stress_home_loss_self	#4363d8
Family Support	stress_family_support	#e6beff
Facebook Friends	parsed_derived_stress_facebk_friends_cat	#008080
Anxiety	stress_anxious	#f58231
Work Outside Home	work_outside_home	#ffe119
Employment Hours	parsed_employment_hours	#f032e6
Tired	stress_tired	#f58231
Stress Past 3 years	stress_past_3yr	#f58231
Stress First 10 Years	stress_first_10yr	#e6beff
Satisfaction with Height	stress_height_satisfy	#ffe119
Other Stress-2	stress_other_experience2	#fffac8
Major Illness	stress_major_illness	#4363d8
Job Loss - Self	stress_job_loss_self	#4363d8
Difference in Desired Rest	diffrest	#bcf60c
Accident	stress_accident	#fffac8
Support System (Binary)	parsed_derived_stress_support_people_bin	#e6beff
Spiritual	stress_spiritual	#3cb44b
Sleep Quality - No Work	stress_sleep_qlty_nowork	#fabebe
Shy	stress_shy	#e6194b
Satisfaction with Weight	stress_body_wt_satisfy	#fabebe
Satisfaction with Appearance	stress_appearance_satisfy	#fabebe
Sad	stress_sad	#f58231

Relationship Stress-5	stress_relationship_status___5	#ffe119	
Preferred Wake Time	difftowake	#9a6324	
Other Stress-1	stress_other_experience1	#fffac8	
Moderate Activity	moderate_activity	#e6194b	
Father of Baby Support	stress_fob_support	#e6194b	
Difference in Desired Wake Time	parsed_timepreftowake	#9a6324	

Supplementary Table 4: *Stress feature questionnaire. Questions asked for each stress feature used in the study. Questions (except for birth "complications" and "defects") are from the DQAQ-SPF Questionnaire, copyright (2020) Firdaus S. Dhabhar and the University of Miami, jointly with Stanford University. All rights reserved. This questionnaire may not be used fully or partially, reproduced, displayed, modified, or distributed without the express prior written permission from Dr. Dhabhar (dhabhar@gmail.com).*

Feature Name	Question
**birth_defect_reason**	If you answered somewhat or much higher, to what do you attribute that risk? Si su repuesta es, algo parecido o mas alto, como concluye y basa Usted esos riesgos? (multiple choice)
**stress_pain**	How often do you experience pain (headache, backache, soreness, etc.)?
**hrsrest**	How many hours of sleep DO YOU NEED TO FEEL RESTED in the morning?
**stress_divorce_self**	Self divorced: YES/NO
**difftobed**	Difference to preferred bed time.
**defect_risk**	Some women may say that their risk for having a baby with a birth defect is low, average or high. Think about yourself compared to most other pregnant women. Using the following scale, would you say that your degree of risk compared to these women is:Algunas mujeres dicen que para ellas el riesgo de dar a luz un bebe prematuramente, un bebe con un defecto de nacimiento es muy bajo, promedio, o alto. Piense acerca de usted misma y comparase con otras mujeres embarazadas. Usando la siguiente escala de medida, Usted diria que el grado de riesgo en comparacion con otras mujeres es: 0-5
**birth_complications_risk**	Some women may say that their risk for having birth complications like an early delivery, low birth weight or pregnancy complications (not birth defects) is low, average or high. Think about yourself compared to most other pregnant women your age. Using the following scale, would you say that your degree of risk compared to these women is: Algunas mujeres dicen que para ellas el riesgo de dar a luz un bebe prematuramente, con un peso menos de lo esperado, o un embarazo con complicaciones (un bebe sin defectos) es muy bajo, promedio, o alto. Piense acerca de usted misma y comparase con otras mujeres de su edad. Usando la siguiente escala de medida, Diria usted que el grado de riesgo suyo en comparacion con otras mujeres es: 0-5
**stress_optimistic**	How OPTIMISTIC are you?
**stress_heath_prev_3yr**	How HEALTHY have you felt for most of the time during the past THREE years?
**stress_current_health**	How HEALTHY do you feel AT THIS MOMENT?
**stress_religious**	How RELIGIOUS are you? (You do NOT need to state your religion.)
**stress_pain_scale**	How intense is the pain?
**stress_depressed**	How DEPRESSED have you been for most of the time during the past THREE years?
**stress_angry**	How ANGRY have you been for most of the time during the past THREE years?
**preg_risk**	Recoded `birth_complications_risk`
**stress_relationship_status___3**	Married
**stress_relationship_status___2**	In relationship
**stress_life_31_40**	How stressful was/is your life between the AGES OF 31 AND 40? (Choose "N/A" if age not applicable)
**stress_home_loss_self**	Loss of own home:
**stress_family_support**	How SUPPORTIVE have your parents and siblings been during the past THREE years?
**parsed_derived_stress_facebk_friends_cat**	If relevant, please state your approximate number of friends on Facebook.
**stress_anxious**	How ANXIOUS would you say you generally are as you go about your day-to-day activities?

**work_outside_home**	Do you presently work outside the home? Esta usted trabajando afuera de su casa?
**parsed_employment_hours**	How many hours per week do you currently work? Cuantas horas por semana usted trabaja en la actualidad? Hrs/week
**stress_tired**	How TIRED/FATIGUED have you been for most of the time during the past THREE years?
**stress_past_3yr**	How stressful have the PAST THREE YEARS been for you?
**stress_first_10yr**	How stressful were the FIRST 10 YEARS of your life?
**stress_height_satisfy**	How SATISFIED have you generally been with your HEIGHT since your late teenage years?
**stress_other_experience2**	Other? YES/NO
**stress_major_illness**	Major Illness?: YES/NO
**stress_job_loss_self**	Loss of own job: YES/NO
**diffrest**	Difference of actual sleep time to the sleep time the mother feels rested.
**stress_accident**	Serious Accident: YES/NO
**parsed_derived_stress_support_people_bin**	Binarized number of support people
**stress_spiritual**	How SPIRITUAL are you?
**stress_sleep_qlty_nowork**	Generally, how good is your quality of sleep the NIGHT BEFORE WEEKENDS AND HOLIDAYS?
**stress_shy**	How SHY are you?
**stress_body_wt_satisfy**	How SATISFIED have you generally been with your BODY WEIGHT since your late teenage years?
**stress_appearance_satisfy**	How SATISFIED have you been with your OVERALL APPEARANCE since your late teenage years?
**stress_sad**	How SAD have you been for most of the time during the past THREE years?
**stress_relationship_status___5**	Remarried
**difftowake**	Difference to preferred wake time.
**stress_other_experience1**	Other? YES/NO
**moderate_activity**	Do you engage in any moderate physical exercise currently? Usted practica algun tipo de ejercicio fisico moderadamente en la actualidad?
**stress_fob_support**	How SUPPORTIVE has the baby's father been during the past ONE year?
**parsed_timepreftowake**	Preferred wake time.
birth_defect_risk	Some women may say that their risk for having a baby with a birth defect is low, average or high. Think about yourself compared to most other pregnant women. Using the following scale, would you say that your degree of risk compared to these women is: Algunas mujeres dicen que para ellas el riesgo de dar a luz un bebe prematuramente, un bebe con un defecto de nacimiento es muy bajo, promedio, o alto. Piense acerca de usted misma y comparase con otras mujeres embarazadas. Usando la siguiente escala de medida, Usted diria que el grado de riesgo en comparacion con otras mujeres es:
hrsget	Average sleep time.
moderate_act_compare	Is this more or less than prior to your current pregnancy? Es estos mas o menos que lo que hacia anteriormente de su estado de embarazo?
parsed_derived_implicit_hrsget	Derived and automatically parsed from \`stress_sleep_avg\`:<br>How many hours of sleep DO YOU GET ON AVERAGE?
parsed_derived_implicit_hrspref	Derived and automatically parsed
parsed_stress_facebk_friends	Number of Facebook friends.
parsed_stress_sleep_prev_night	Sleep time the previous night.
parsed_stress_support_people	Number of support people
parsed_timegentobed	
parsed_timehavetowake	Imposed bedtime
parsed_timepreftobed	Preferred bedtime
stress_ages_11_20	How stressful was/is your life between the AGES OF 11 AND 20?
stress_b4_kindergrtn	Before kindergarten, did you go to: Preschool, Daycare, neither
stress_bully	Bullying
stress_death_lovd1	Death of loved one: YES/NO
stress_divorce_parent	Parents divorced: YES/NO

stress_glasses_use	Do you use glasses/contacts for: multiple choice
stress_handedness	Do you write with your: left/right/both
stress_happy	How HAPPY have you been for most of the time during the past THREE years?
stress_home_loss_parent	Loss of parents' home:
stress_job_loss_parent	Loss of parents' job
stress_life_21_30	How stressful was/is your life between the AGES OF 21 AND 30? (Choose "N/A" if age not applicable)
stress_life_41_50	How stressful was/is your life between the AGES OF 41 AND 50? (Choose "N/A" if age not applicable)
stress_lonely	How LONELY have you been for most of the time during the past THREE years?
stress_past_1yr	How stressful has the PAST ONE YEAR been for you?
stress_pessimistic	How PESSIMISTIC are you?
stress_quest_complete	How stressful was it to fill out this questionnaire?
stress_relationship_status___1	Single
stress_relationship_status___4	Divorced
stress_sleep_qlty_work	Generally, how good is your quality of sleep the NIGHT BEFORE WORKING DAYS?
vigorous_act_compare	Is this more or less than prior to your current pregnancy? Es esta cantidad de ejercicios lo mismo o menos de lo que Usted hacia antes de su estado de embarazo?
vigorous_activity	Do you engage in any vigorous physical exercise currently? Practica algun tipo de ejercicios fisicos vigorosamente en la actualidad?
birth_complication_reason	If you answered somewhat or much higher, to what do you attribute that risk? Si su repuesta es, algo parecido o mas alto, como concluye y basa Usted esos riesgos?
